# Heterologous redox partners supporting the efficient catalysis of epothilone B biosynthesis by EpoK in *Schlegelella brevitalea*

**DOI:** 10.1186/s12934-020-01439-5

**Published:** 2020-09-15

**Authors:** Junheng Liang, Huimin Wang, Xiaoying Bian, Youming Zhang, Guoping Zhao, Xiaoming Ding

**Affiliations:** 1 Collaborative Innovation Center for Genetics and Development, State Key Laboratory of Genetic Engineering, Department of Microbiology, School of Life Sciences, Fudan University, Shanghai, 200438 People’s Republic of China; 2 Shandong University-Helmholtz Institute of Biotechnology, State Key Laboratory of Microbial Technology, School of Life Sciences, Shandong University, Qingdao, Shandong People’s Republic of China; 3 CAS Key Laboratory of Synthetic Biology, Institute of Plant Physiology and Ecology, Shanghai Institutes for Biological Sciences, Chinese Academy of Sciences, Shanghai, People’s Republic of China

**Keywords:** Epothilone B, EpoK (CYP167A1), Electron transport partner, Gene overexpression, Whole-cell biotransformation

## Abstract

**Background:**

Epothilone B is a natural product that stabilizes microtubules, similar to paclitaxel (Taxol); therefore, epothilone B and several derivatives have shown obvious antitumour activities. Some of these products are in clinical trials, and one (ixabepilone, BMS) is already on the market, having been approved by the FDA in 2007. The terminal step in epothilone B biosynthesis is catalysed by the cytochrome P450 enzyme EpoK (CYP167A1), which catalyses the epoxidation of the C12–C13 double bond (in epothilone C and D) to form epothilone A and B, respectively. Although redox partners from different sources support the catalytic activity of EpoK in vitro, the conversion rates are low, and these redox partners are not applied to produce epothilone B in heterologous hosts.

**Results:**

*Schlegelella brevitalea* DSM 7029 contains electron transport partners that efficiently support the catalytic activity of EpoK. We screened and identified one ferredoxin, Fdx_0135, by overexpressing putative ferredoxin genes in vivo and identified two ferredoxin reductases, FdR_0130 and FdR_7100, by whole-cell biotransformation of epothilone C to effectively support the catalytic activity of EpoK. In addition, we obtained strain H7029-3, with a high epothilone B yield and found that the proportion of epothilone A + B produced by this strain was 90.93%. Moreover, the whole-cell bioconversion strain 7029-10 was obtained; this strain exhibited an epothilone C conversion rate of 100% in 12 h. Further RT-qPCR experiments were performed to analyse the overexpression levels of the target genes. Gene knock-out experiments showed that the selected ferredoxin (Fdx_0135) and its reductases (FdR_0130 and FdR_7100) might participate in critical physiological processes in DSM 7029.

**Conclusion:**

Gene overexpression and whole-cell biotransformation were effective methods for identifying the electron transport partners of the P450 enzyme EpoK. In addition, we obtained an epothilone B high-yield strain and developed a robust whole-cell biotransformation system. This strain and system hold promise for the industrial production of epothilone B and its derivatives.

## Introduction

Epothilones are microtubule-stabilizing antitumour compounds with a mechanism similar to that of paclitaxel; they were initially discovered to be produced by the myxobacterium *Sorangium cellulosum* approximately 30 years ago [1]. Epothilone A and epothilone B were the first two isolated metabolites. Due to its higher binding affinity for tubulin, epothilone B was proven to be more active than epothilone A and even paclitaxel [2]. Moreover, this series of compounds has the advantages of increased water solubility and the capacity for large-scale production through bacterial fermentation [3]. Currently, epothilone B and its derivatives are undergoing multiple clinical trials for the treatment of malignant tumours [4], and ixabepilone, a lactam derivative of epothilone B, was approved by the FDA for the clinical treatment of advanced breast cancer in 2007 [5]. Recently, some studies of epothilone B have focused on neurological diseases because epothilones have a strong ability to stabilize microtubules and penetrate the blood-brain barrier [6]. Epothilone B can promote axon regeneration after spinal cord injury, protect nigral dopaminergic neurons in the mouse lesion model of Parkinson’s disease induced by 6-hydroxydopamine, and accelerate corneal nerve regrowth and functional recovery. These studies indicate that epothilone B hold promise for clinical use in some neurological diseases [7–9].

The production of epothilone has historically been a limiting factor in its application. The original producer *S. cellulosum* is used for industrial synthesis of epothilone B, but this strain has many disadvantages. Since its discovery, epothilone B has been synthesized by total synthesis, semisynthesis, and chemical modification strategies because its chemical structure is relatively simple [10]. However, because of their multi-step reaction sequences, low yield, and high cost, these strategies cannot meet the needs of industrial production. Several different sources of epothilone biosynthetic gene clusters have been sequenced and identified [2, 11, 12]. Epothilone synthesis via transformation of the epothilone gene cluster into *Myxococcus xanthus*, *Escherichia coli*, *Streptomyces coelicolor*, *Streptomyces venezuelae*, *Pseudomonas putida*, and *Schlegelella brevitalea* (DSM 7029) has been attempted over the past few years [13–18].

In most heterologous epothilone expression systems, EpoK, a cytochrome P450 enzyme responsible for the conversion of epothilone C/D to epothilone A/B, respectively, by C12–C13 epoxidation, is deleted or inactivated. These systems cannot produce epothilone B but instead produce its precursor epothilone D [2, 13–16]. In 2003, the crystal structure of EpoK was analysed; it exhibits a typical triangular prism-shaped P450 fold similar to that of other P450s (Additional file 1: Figure S1) [19]. Cytochrome P450 enzymes catalyse monooxygenation reactions by inserting one oxygen atom from O_2_ into specific substrates, and this process requires the consecutive delivery of two electrons derived from NAD(P)H via redox partner proteins to the ferric P450 [20]. Most bacterial cytochrome P450 systems are class I P450 systems, and class I cytochrome P450 enzymes often use ferredoxin reductases and ferredoxins as electron mediators [21]. In summary, we inferred that EpoK most likely uses the class I electron transfer system, which normally consists of an iron-sulfur ferredoxin and an NAD(P)H-dependent ferredoxin reductase.

The identification of autologous natural redox partners of EpoK is relatively complicated because the genes encoding potential redox partners are not located near the epothilone gene cluster, and the genome of *S. cellulosum* encodes many oxidoreductases. Therefore, surrogate redox partners are employed to reconstitute the catalytic activity of EpoK in vitro. In 2000, Bryan Julien et al. observed a conversion rate of epothilone D to epothilone B of 82% with an epothilone D concentration of 100 mM using the redox proteins from spinach [2], but Kern et al. failed to replicate these results. They investigated homo- and heterologous electron transport systems for the in vitro conversion of epothilone D by EpoK and found that the hybrid redox system containing ferredoxin from *Synechocystis* and ferredoxin-NADP^+^ reductase (FNR) from *Chlamydomonas reinhardtii* (EpoK:Fdx:FNR ratio 1:20:3) showed the fastest conversion rate, and the utilization of the homologous redox partners Fdx8/FdR_B from *S. cellulosum* So ce56 for EpoK production resulted in the highest conversion rate of epothilone D (74.4%) [22]. These surrogate redox partners have not been applied in the heterologous expression system for the production of epothilone B, and whether they can deliver electrons efficiently in vivo is still unknown.

In our previous work, we reported the high-yield production of epothilone C and D in the heterologous host DSM 7029 [23]. Based on this heterologous expression platform, we found that there were redox partners from DSM 7029 to support the activity of EpoK. We also screened and identified two pairs of EpoK-supporting ferredoxin/ferredoxin reductase from DSM 7029 by the overexpression of putative ferredoxin genes and whole-cell biotransformation of epothilone C. Therefore, through a one-step fermentation process with strain H7029-3, we obtained a high yield of epothilone B, with a proportion of epothilone A + B as high as 90.93% (the yield of epothilone B was 35.787 mg L^−1^). In addition, an epothilone C whole-cell biotransformation system was constructed, and the maximum conversion rate of 100% was attained in 12 h.

## Results

### A large amount of epothilone A and epothilone B was detected when *epoK* was overexpressed in the epothilone C/D high-yield strain H7029-1

**Fig. 1:**
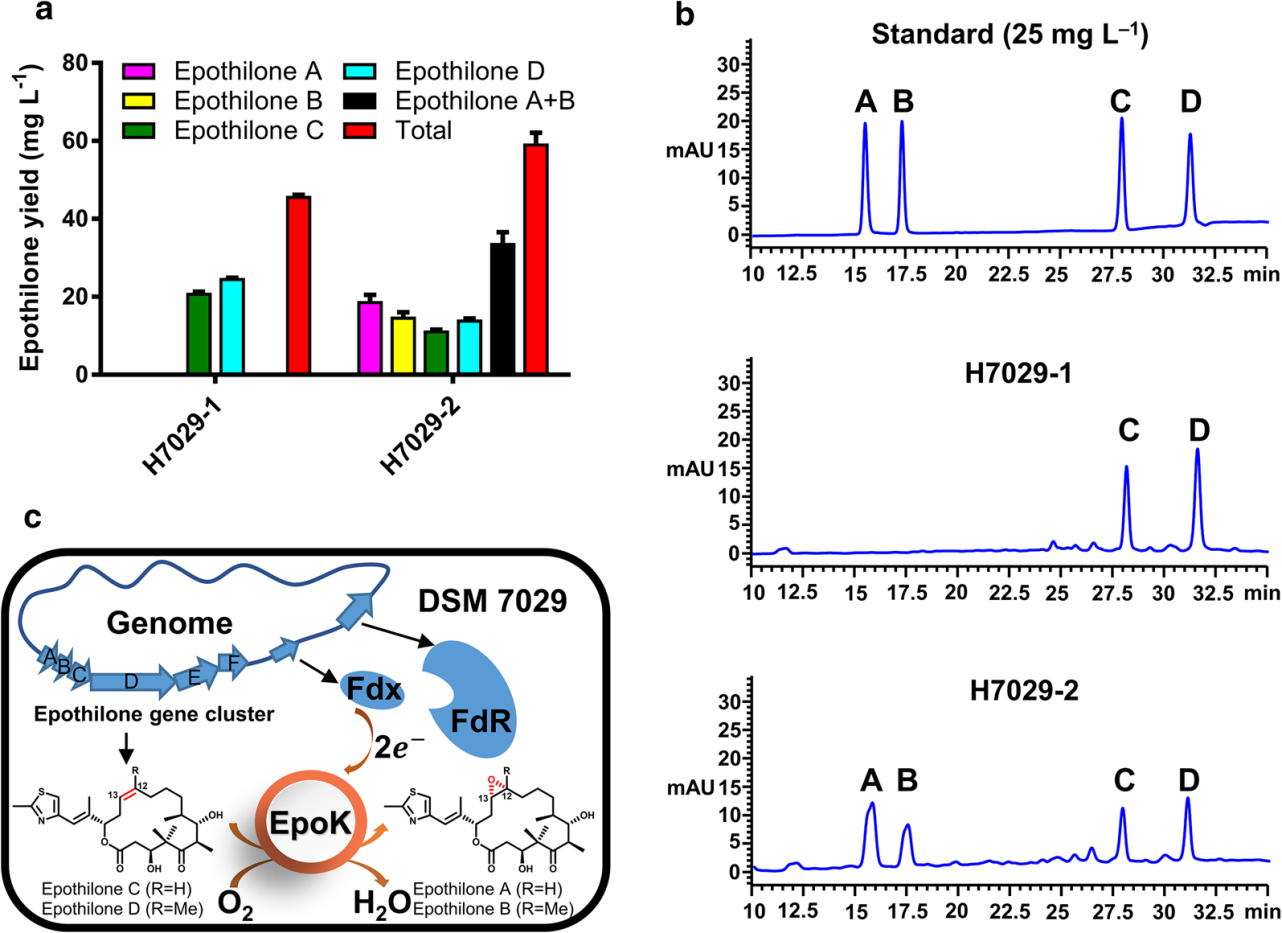
Highly efficient redox proteins support the catalytic activity of EpoK in DSM 7029. a Epothilone yield of strain H7029-2 with *epoK* expression. The data are presented as the means ± standard deviations of the values from three independent experiments (n = 3). b HPLC analysis of the standard sample (25 mg L^−1^) and the extracts from the parental strain H7029-1 and the mutant strain H7029-2. A, epothilone A; B, epothilone B; C, epothilone C; D, epothilone D. c Schematic diagram: EpoK efficiently catalysed the conversion of epothilone C and D to epothilone A and B, respectively, in the epothilone C/D high-yield strain H7029-1. The epothilone gene cluster from *S. cellulosum* So0157-2 was transposed into the genome of the heterologous host DSM 7029

In our previous study, when the entire epothilone cluster of *S. cellulosum* So0157-2 or So ce90 was introduced into *S. brevitalea* DSM 7029 by transposition, the resulting strains MMR1001 and G32 produced small amounts of epothilone A and epothilone B [18, 23]. Furthermore, we constructed the *epoK*-overexpressing strain H7029-2, which contained the plasmid oriV-trfA-Apra^r^-P_*kan*_-*epoK* with the constitutive *Tn5*-*kan* promoter (P_*kan*_) from the previously reported epothilone C/D high-yield strain H7029-1 (epothilone C + epothilone D = 46 mg L^−1^). Epothilone A and epothilone B were detected in the fermentation products of H7029-2 (epothilone A + epothilone B = 33.857 ± 4.636 mg L^−1^), and the proportion of epothilone A + B was 56.87 ± 3.23% (Fig. 1a, b), whereas the EpoK-deficient strain H7029-1 did not produce either epothilone A or epothilone B. These results indicated that DSM 7029 did not express a P450 enzyme capable of epoxidizing epothilone C or D but did express suitable redox partners to support the catalytic activity of EpoK (Fig. 1c).

### Screening and identification of effective ferredoxins for EpoK by overexpressing the putative ferredoxin genes from DSM 7029

Searching the genome of strain DSM 7029 for possible electron transport partners of EpoK revealed six putative ferredoxin genes and two putative ferredoxin reductase genes. All ferredoxin genes were scattered on the chromosome and were not located near any of the putative ferredoxin reductase genes or either of the two cytochrome P450 genes in DSM 7029, except the ferredoxin gene *fdx_0135*, which was adjacent to the ferredoxin reductase gene *fdr_0130* (Additional file 1: Figure S2). Analysis of the sequences and structures of these proteins indicated that ferredoxins Fdx_2170, Fdx_4560, and Fdx_6105 were [2Fe-2S] ferredoxins, Fdx_0135 was a hybrid [3Fe-4S]/[4Fe-4S] ferredoxin, and Fdx_2730 and Fdx_5185 were [4Fe-4S] ferredoxins (Table 1). The ferredoxin reductases FdR_0130 and FdR_7100 were ferredoxin-NADP reductases (Table 2). Previously, Kern et al. demonstrated that the EpoK/Fdx8/FdR_B system from *S. cellulosum* So ce56 efficiently converted epothilone D in vitro [22]. Sequence analysis of FdR_B and Fdx_8 revealed high identity (92.7% and 78.2%) to the putative ferredoxin reductase *fdr_S2240* and the ferredoxin *fdx_S4580* from *S. cellulosum* So0157-2, respectively. In this study, our EpoK was encoded in the genome of *S. cellulosum* So0157-2, and analysis of the amino acid sequences of EpoK from these three sources showed differences in seven amino acids (Additional file 1: Figure S3). Therefore, *fdx_S4580* and *fdr_S2240* were also investigated. The general characteristics of the related components are listed in Table 1 and Table 2.

**Table 1:** Detailed information on the selected ferredoxins

Gene locus_tag	Gene name	Origin	Genome annotation	UniProt entry	No. of amino acids	Fe-S cluster type	Conserved motif
*AAW51_RS10135*	*fdx_0135*	*S. brevitalea* DSM 7029	Ferredoxin family protein	A0A0G3BH78	107	Fe_7_S_8_	CX_7_C and CX_2_CX_2_CX_3_CP
*AAW51_RS22170*	*fdx_2170*	*S. brevitalea* DSM 7029	Rieske 2Fe-2S domain-containing protein	A0A0G3BNW5	128	Fe_2_S_2_	Not conserved
*AAW51_RS22730*	*fdx_2730*	*S. brevitalea* DSM 7029	Ferredoxin family protein	A0A0G3BTB5	80	Fe_4_S_4_	CX_2_CX_2_CX_n_CP
*AAW51_RS04560*	*fdx_4560*	*S. brevitalea* DSM 7029	(2Fe-2S) ferredoxin	A0A0G3BJP5	108	Fe_2_S_2_	Not conserved
*AAW51_RS05185*	*fdx_5185*	*S. brevitalea* DSM 7029	YfhL family 4Fe-4S dicluster ferredoxin	A0A0G3BME9	109	Fe_4_S_4_	CX_2_CX_2_CX_n_CP
*AAW51_RS16105*	*fdx_6105*	*S. brevitalea* DSM 7029	ISC system 2Fe-2S type ferredoxin	A0A0G3BPK5	112	Fe_2_S_2_	CX_5_CX_2_CX_35-37_C
*SCE1572_RS34580*	*fdx_S4580*	*S. cellulosum* So0157-2	4Fe-4S dicluster domain-containing protein	None	102	Fe_7_S_8_	CX_7_C and CX_2_CX_2_CX_3_CP
*DEH84_S06445*	*fdx_A6445*	*A. olei* NBRC 110486	Ferredoxin	A0A2U8FPX2	107	Fe_7_S_8_	CX_7_C and CX_2_CX_2_CX_3_CP

**Table 2:** Detailed information on the selected ferredoxin reductases

Gene locus_tag	Gene name	Origin	Genome annotation	UniProt entry	No. of amino acids	Classification
*AAW51_RS10130*	*fdr_0130*	*S. brevitalea* DSM 7029	NAD(P)/FAD-dependent oxidoreductase	A0A0G3BMU8	372	Bacterial-type
*AAW51_RS27100*	*fdr_7100*	*S. brevitalea* DSM 7029	Ferredoxin-NADP reductase	A0A0G3BRU1	257	Bacterial-type
*SCE1572_RS32240*	*fdr_S2240*	*S. cellulosum* So0157-2	Ferredoxin family protein	A0A0G3BTB5	245	Bacterial-type

**Fig. 2:**
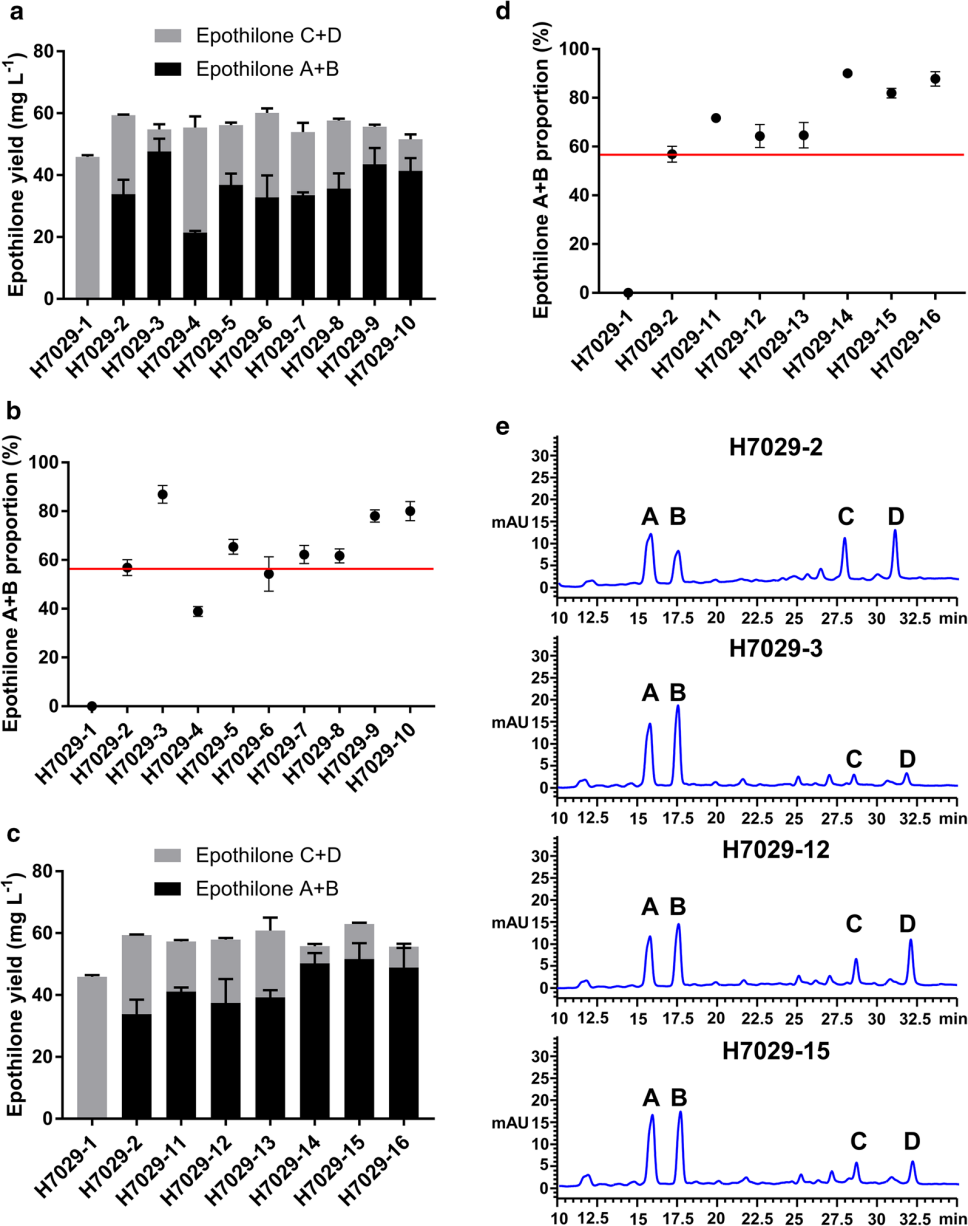
Screening and identification of EpoK-supporting redox partners through the overexpression of putative ferredoxin and ferredoxin reductase genes. **a** Epothilone A + B and the total production of the parental strain H7029-1, H7029-2 strain expressing *epoK,* and strains H7029-3 to H7029-10 overexpressing eight other selected ferredoxin genes. **b** Comparison of the epothilone A + B proportions in the strains overexpressing the putative ferredoxin genes. **c** Epothilone A + B and the total production of the parental strain H7029-1, the H7029-2 strain expressing *epoK,* and strains H7029-11 to H7029-16 overexpressing three other putative ferredoxin reductase genes alone or in combination with *fdx_0135* or *fdx_S4580*. **d** Comparison of the epothilone A + B proportions in the strains overexpressing the putative ferredoxin reductase genes alone or in combination with *fdx_0135* or *fdx_S4580*. **e** HPLC analysis of the extracts from strains H7029-2, H7029-3, H7029-12, and H7029-15. The changes in the composition of epothilone clearly represented the results of overexpressing ferredoxin and ferredoxin reductase genes. A, epothilone A; B, epothilone B; C, epothilone C; D, epothilone D. All strains above were analysed in triplicate, and the data are presented as the means ± standard deviations of the values from three independent experiments (n = 3)

To isolate and identify highly efficient electron transport partners of EpoK, all ferredoxin and ferredoxin reductase genes were overexpressed under the control of the P_*kan*_ promoter, and their sequences were cloned into the plasmid oriV-trfA-Apra^r^-P_*kan*_-*epoK*-P_*kan*_-*fdx/fdr* (to generate pEOK103 to pEOK116). Then, these plasmids were transformed into H7029-1 by electroporation, and colony PCR was used to check the colonies grown on the selection plate to verify these transformants (Additional file 1: Figure S4). These engineered strains of H7029-1 are listed in Table 3. The fermentation products were detected by high-performance liquid chromatography (HPLC), and all strains overexpressing ferredoxins or ferredoxin reductases produced epothilone A, B, C and D. Among the strains overexpressing putative ferredoxin genes, H7029-3 and H7029-9 exhibited obviously higher conversion rates than H7029-2, producing proportions of epothilone A + B of 86.91% ± 3.62% and 78.05% ± 2.52%, respectively (Additional file 1: Table S1). In contrast, the conversion rate of the other strains with overexpression of the other five ferredoxin genes was barely increased. Coincidentally, the two effective ferredoxins, *fdx_0135* and *fdx_S4580*, were hybrid [3Fe-4S]/[4Fe-4S] ferredoxins. In addition, we tested *fdx_A6445* from the *Aquabacterium olei* strain NBRC 110486, which shared 87.46% nucleic acid identity with *fdx_0135* and was also predicted to be a hybrid [3Fe-4S]/[4Fe-4S] ferredoxin (Additional file 1: Figure S5). Strain H7029-10 with the overexpression of *fdx_A6445* also produced a greatly increased proportion of epothilone A + B (Fig. 2a, b).

**Table 3:** List of engineered strains of DSM 7029 used in this study

Classification	Strain	Relevant genotype/property	Source
Redox gene overexpression	H7029-1	Epothilone C/D high-yield strain	This study
	H7029-2	H7029-1-*epoK*	This study
	H7029-3	H7029-1-*epoK*-*fdx_0135*	This study
	H7029-4	H7029-1-*epoK*-*fdx_2170*	This study
	H7029-5	H7029-1-*epoK*-*fdx_2730*	This study
	H7029-6	H7029-1-*epoK*-*fdx_4560*	This study
	H7029-7	H7029-1-*epoK*-*fdx_5185*	This study
	H7029-8	H7029-1-*epoK*-*fdx_6105*	This study
	H7029-9	H7029-1-*epoK*-*fdx_S4580*	This study
	H7029-10	H7029-1-*epoK*-*fdx_A6445*	This study
	H7029-11	H7029-1-*epoK*-*fdr_0130*	This study
	H7029-12	H7029-1-*epoK*-*fdr_7100*	This study
	H7029-13	H7029-1-*epoK*-*fdr_S2240*	This study
	H7029-14	H7029-1-*epoK*-*fdx_0135*-*fdr_0130*	This study
	H7029-15	H7029-1-*epoK*-*fdx_0135*-*fdr_7100*	This study
	H7029-16	H7029-1-*epoK*-*fdx_S4580*-*fdr_S2240*	This study
Whole-cell biotransformation	7029-1	DSM 7029 derivate, harbouring pEOK101, DSM 7029-empty vector	This study
	7029-2	DSM 7029 derivate, harbouring pEOK102, DSM 7029-*epoK*	This study
	7029-3	DSM 7029 derivate, harbouring pEOK10, DSM 7029-*epoK*-*fdx_0135*	This study
	7029-4	DSM 7029 derivate, harbouring pEOK109, DSM 7029-*epoK*-*fdx_S4580*	This study
	7029-5	DSM 7029 derivate, harbouring pEOK11, DSM 7029-*epoK*-*fdr_0130*	This study
	7029-6	DSM 7029 derivate, harbouring pEOK112, DSM 7029-*epoK*-*fdr_7100*	This study
	7029-7	DSM 7029 derivate, harbouring pEOK113, DSM 7029-*epoK*-*fdr_S2240*	This study
	7029-8	DSM 7029 derivate, harbouring pEOK114, DSM 7029-*epoK*-*fdx_0135*-*fdr_0130*	This study
	7029-9	DSM 7029 derivate, harbouring pEOK115, DSM 7029-*epoK*-*fdx_0135*-*fdr_7100*	This study
	7029-10	DSM 7029 derivate, harbouring pEOK116, DSM 7029-*epoK*-*fdx*_*S4580-fdr*_*S2240*	This study
Redox gene knock-out	JH01	H7029-14 derivate, *fdx_0135* knock-out	This study
	JH02	H7029-14 derivate, *fdr_0130* knock-out	This study
	JH03	H7029-12 derivate, *fdr_7100* knock-out	This study

When the three ferredoxin reductase genes were separately overexpressed in H7029-2, all three resulting strains, H7029-11, H7029-12, and H7029-13, had slightly higher conversion rates than H7029-2 (71.71% ± 0.79%, 64.33% ± 4.69%, and 64.66% ± 5.18%, respectively) (Fig. 2c, d; Additional file 1: Table S1). Further experiments were conducted by co-expressing the effective gene *fdx_0135* with *fdr_0130* or *fdr_7100* and co-expressing the other effective gene *fdx_S4580* together with the endogenous *fdr_S2240*. The proportions of epothilone A + B produced by these three strains (H7029-14, H7029-15, and H7029-16) did not significantly exceed the proportions produced by strains H7029-3 and H7029-9 (Fig. 2c, d; Additional file 1: Table S1). HPLC analysis results of the extracts from strains H7029-2, H7029-3, H7029-12, and H7029-15 clearly showed changes in the epothilone products when the ferredoxin or ferredoxin reductase genes were overexpressed (Fig. 2e). In summary, we successfully screened two ferredoxins to support the catalytic activity of EpoK by overexpressing putative ferredoxin genes in the epothilone C/D high-yield strain H7029-1, but when the putative ferredoxin reductase genes were overexpressed separately or co-overexpressed with the effective ferredoxin genes by the same method, the proportion of epothilone A + B was not apparently increased. We hypothesized that the ferredoxin reductase was saturated under this condition.

### Identification of ferredoxin reductases for EpoK by epothilone C whole-cell biotransformation

**Fig. 3:**
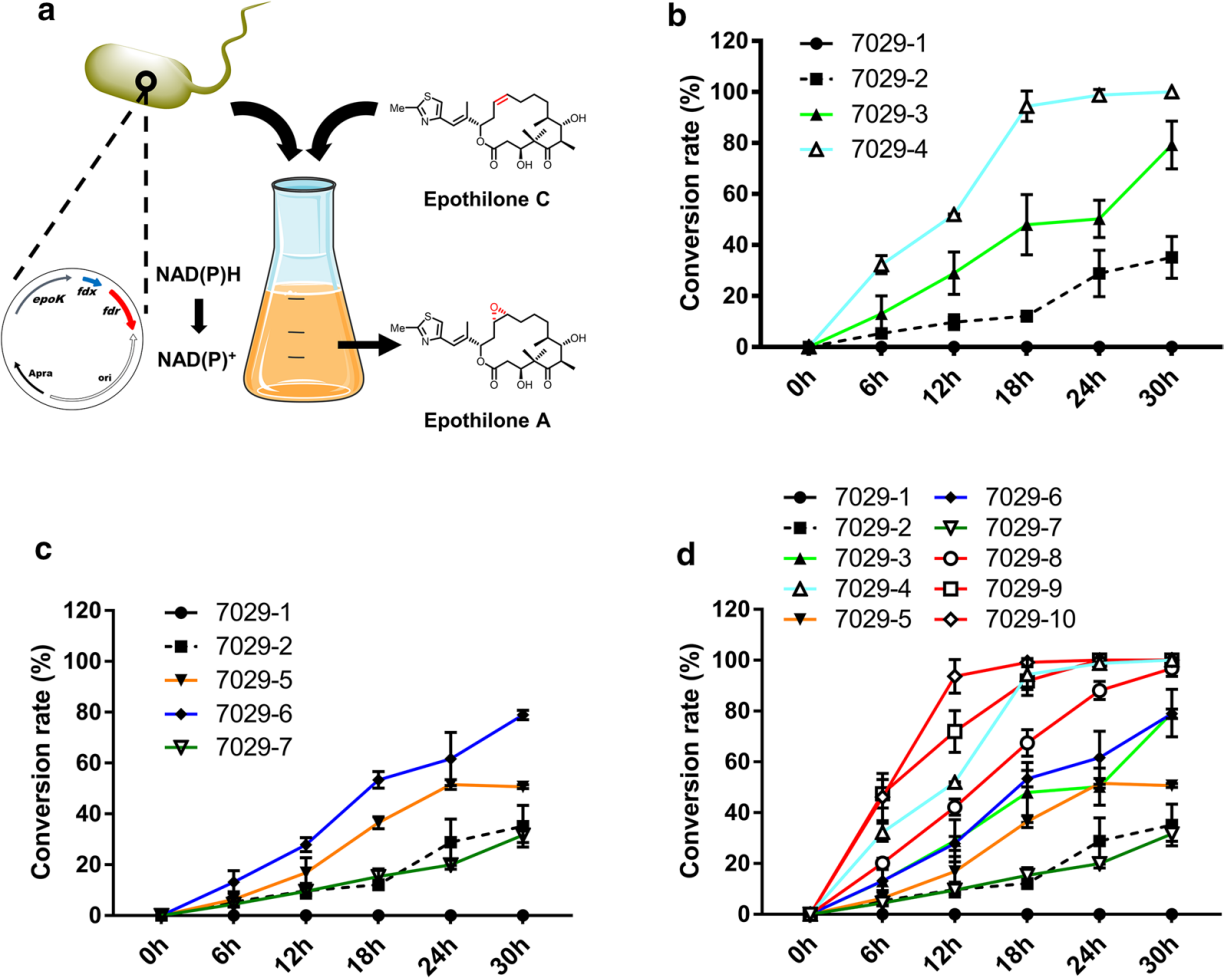
Whole-cell biotransformation of epothilone C with engineered DSM 7029 to screen for EpoK-supporting ferredoxin reductases. **a** Schematic diagram: the whole-cell biotransformation system catalysed the conversion of epothilone C to epothilone A. **b** Epothilone C conversion rates of the *fdx_0135* and *fdx_S4580* overexpression strains at different reaction times (6 h, 12 h, 18 h, 24 h, and 30 h). Strain 7029-1 is DSM 7029 transformed with empty vector; strain H7029-2 is DSM 7029 with *epoK* expression. **c** Epothilone C conversion rates of strains overexpressing three ferredoxin reductase genes at different reaction times (6 h, 12 h, 18 h, 24 h, and 30 h). **d** Epothilone C conversion rates of strains overexpressing ferredoxin genes or ferredoxin reductase genes alone or in combination at different reaction times (6 h, 12 h, 18 h, 24 h, and 30 h). All strains above were analysed in triplicate, and the data are presented as the means ± standard deviations of the values from three independent experiments (n = 3)

In the heterologous expression system H7029-2 with *epoK* expression, epothilone C and D were gradually synthesized and then epoxidated by EpoK to produce epothilones A and B, respectively. Under these conditions, the catalytic potential of EpoK was only partially utilized; thus, there was little demand for electron transport partners, especially ferredoxin reductase. To evaluate the catalytic activity of EpoK with identified ferredoxins and putative ferredoxin reductases in the presence of high substrate concentrations, we designed an EpoK whole-cell biocatalyst based on the wild-type strain DSM 7029 and applied it for epothilone C conversion (a large amount of epothilone C crude extract was prepared in our laboratory; Additional file 1: Figure S6). Furthermore, we sought to determine whether we could use this whole-cell biocatalyst system to identify effective ferredoxin reductases to support the catalytic activity of EpoK (Fig. 3a).

Ten mutants (7029-1 to 7029-10, Table 3) were constructed by transformation of the corresponding plasmids, and colony PCR was used to check the resistant colonies to confirm these transformants (Additional file 1: Figure S7). Then, we added epothilone C to a concentration of 40 mg L^−1^ in the 50-mL whole-cell biocatalyst system when these mutants were in the late mid-exponential growth phase at approximately 24 h. Surprisingly, all whole-cell systems were able to convert epothilone C to epothilone A in simple CYMG medium. However, the *epoK*-expressing strain 7029-2 converted only approximately 35.18% ± 8.19% of epothilone C after 30 h. Two strains overexpressing effective ferredoxin genes, 7029-3 and 7029-4, converted approximately 79.20% ± 9.32% and 100%, respectively, after 30 h, which were much higher percentages than converted by 7029-2. These two ferredoxins were proven again to efficiently support the activity of EpoK, but under these conditions, Fdx_S4580 was more efficient than Fdx_0135, unlike in the previous experiment (Fig. 3b). Surprisingly, the overexpression of *fdr_0130* and *fdr_7100* promoted the conversion of epothilone C, and the conversion rates of strains 7029-5 and 7029-6 were higher than that of strain 7029-2. However, strain 7029-7 with *fdr_S2240* overexpression, did not have a higher conversion rate than strain 7029-2; thus, we inferred that DSM 7029 did not express a ferredoxin that interacted with FdR_S2240 to transfer electrons for EpoK (Fig. 3c). Further results of *fdx_0135*/*fdr_0130*, *fdx_0135*/*fdr_7100*, and *fdx_S4580*/*fdr_S2240* co-overexpression demonstrated that FdR_0130 and FdR_7100 delivered electrons to Fdx_0135 and that electrons were then transferred to EpoK to complete the catalytic reaction; the conversion rates of strains 7029-8 and 7029-9 were much higher than those of 7029-3, 7029-5, and 7029-6. Surprisingly, the conversion rate of strain 7029-8 was 96.73% in 30 h, and the conversion rate of strain 7029-9 was 100% in 24 h. Moreover, FdR_S2240 delivered electrons to Fdx_S4580, and the conversion rate of strain 7029-10, with *fdx_S4580*/*fdr_S2240* overexpression, was 100% in 12 h (Fig. 3d). In summary, three pairs of electron transfer partners were screened and identified, two of which were from DSM 7029.

### Validation of the transcription levels of *fdx_0135*, *fdr_0130*, and *fdr_7100* by quantitative reverse transcription–polymerase chain reaction (RT-qPCR)

**Fig. 4:**
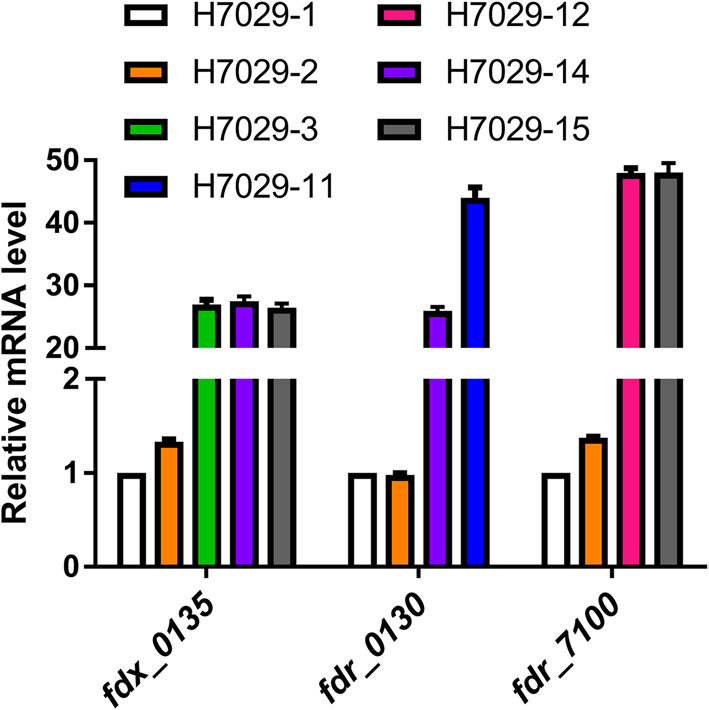
RT-qPCR analysis of the transcription levels of *fdx_0135*, *fdr_0130,* and *fdr_7100*

The above results indicated that *fdx_0135*, *fdr_0130*, and *fdr_7100* overexpression obviously promoted the catalytic activity of EpoK. To understand the relationship between the increase in the transformation efficiency of EpoK and the changes in the transcription levels of these three genes, we used RT-qPCR to analyse the transcription levels of these three genes. Total RNA was extracted from the seven targeted strains harvested in mid-exponential phase, at approximately 18 h, using a total RNA extraction kit. cDNA was then prepared for qPCR. Compared with the corresponding genes in the control strain, *fdx_0135* and *fdr_7100* expressed by plasmids under the control of the P_*kan*_ promoter were upregulated approximately 25- and 47-fold, respectively, whether overexpressed alone or together. When *fdr_0130 was* overexpressed alone under the control of the P_*kan*_ promoter, it was upregulated approximately 44-fold. However, when *fdr_0130* was co-overexpressed with *fdx_0135* in an operon under the control of a single P_*kan*_ promoter, similar to its configuration in the genome, its expression level was 26-fold higher than that in the control strain (Fig. 4). The above RT-qPCR analysis indicated that the transcription levels of *fdx_0135*, *fdr_0130*, and *fdr_7100* were significantly increased, and the catalytic activity of EpoK was thus promoted.

### Knock-out of the *fdx_0135*, *fdr_0130*, and *fdr_7100* genes via plasmid-mediated complementation

**Fig. 5:**
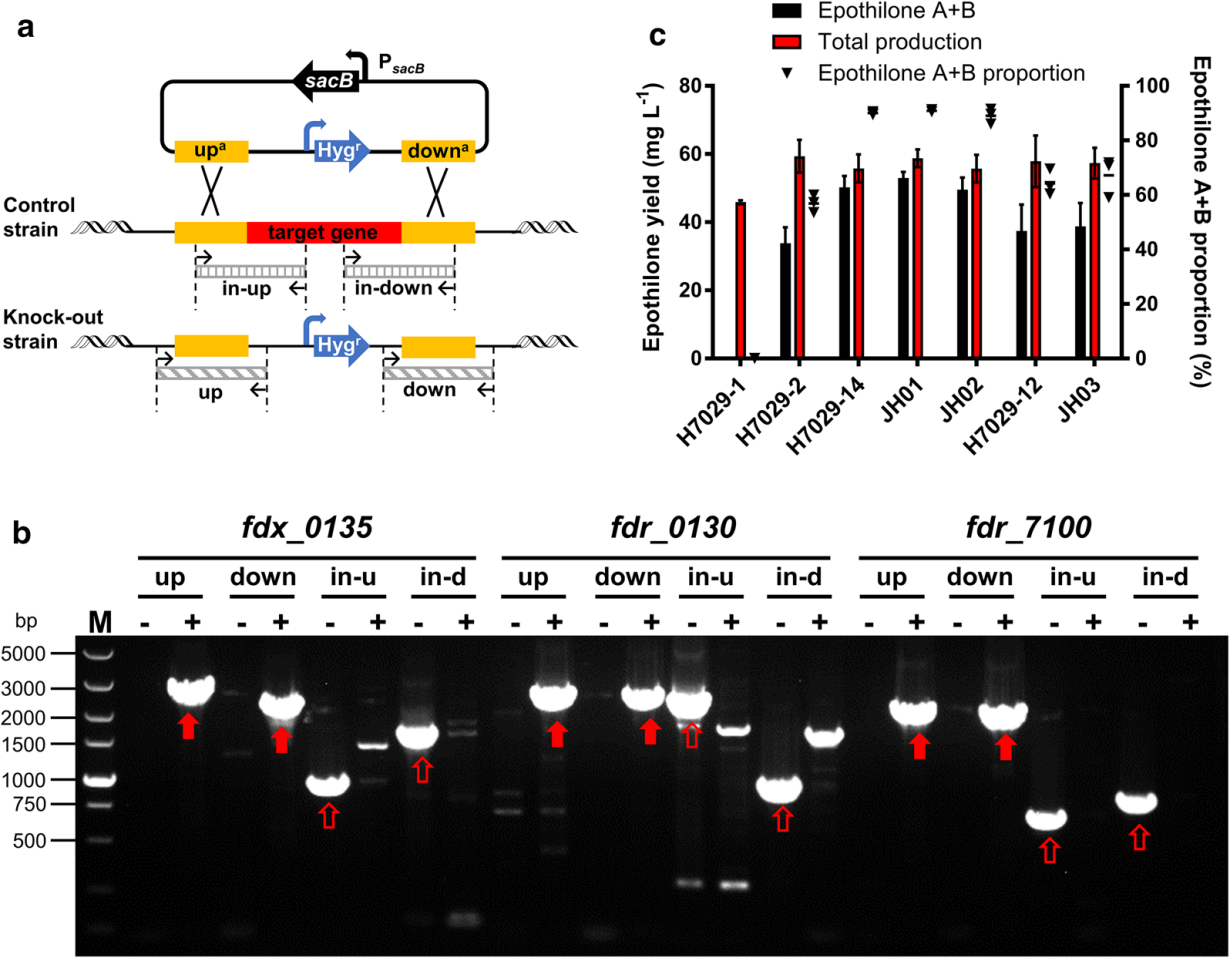
Verification of *fdx_0135*, *fdr_0130* and *fdr_7100* knock-out. **a** Schematic diagram: PCR verification of the knock-out of three redox protein genes. The control strains contained the plasmid expressing each target gene. Knock-out of each target gene was confirmed based on positive and negative PCR verification. The upstream and downstream fragments flanking the left and right homologous arms of the knock-out gene were successfully amplified from the knock-out strains, but the internal fragments near the upstream and downstream homologous arms were amplified from only the control strains (up^a^, upstream homologous arm; down^a^, downstream homologous arm; up, the upstream fragments flanking the upstream homologous arm; down, the downstream fragments flanking the downstream homologous arm; in-up, internal validation sequence near the upstream homologous arm; in-down, internal validation sequence near the downstream homologous arm). **b** One-step selection method for knock-out of the three target genes. Hygromycin and sucrose were added to the electroporation plate for *sacB*-mediated negative selection. Positive PCR verification was conducted by successfully amplifying the up and down fragments of each gene in the knock-out strains; negative PCR verification was conducted by successfully amplifying the in-up and in-down fragments of each gene in the control strains. For *fdx_0135*: up, 2705 bp; down, 2319 bp; in-up, 931 bp; in-down, 1613 bp. For *fdr_0130*: up, 2490 bp; down, 2434 bp; in-up, 2334 bp; in-down, 936 bp. For *fdr_7100*: up, 2174 bp; down, 2106 bp; in-up, 665 bp; in-down, 797 bp. (in-u, in-up; in-d, in-down. PCR fragments from positive PCR verification are marked with solid red arrows; PCR fragments from negative PCR verification are marked with unfilled red arrows). **c** Epothilone A + B production, total production, and epothilone A + B proportion of the strains with *fdx_0135*, *fdr_0130*, and *fdr_7100* knock-out compared with the respective control strains. Each strain was analysed in triplicate, and the data are presented as the means ± standard deviations of the values from three independent experiments (n = 3)

Knocking out redox protein genes is a common strategy to identify natural electron transport partners of a cytochrome P450 enzyme. Therefore, we tried to knock out *fdx_0135*, *fdr_0130*, and *fdr_7100* in strain H7029-1 to confirm whether only these three genes were involved in the epoxidase activity of EpoK. Knock-out plasmids were constructed by homologous recombination, and knock-out mutants were obtained by positive selection with antibiotic resistance genes and negative selection with the *sacB* gene, as described by Lei et al. [24]. However, no colonies were obtained. The two-step double-crossover homologous recombination method showed that these three genes could not be knocked out, considering *fdx_0135* as an example (Additional file 1: Figure S8). Further knock-out experiments were conducted in H7029-14 and H7029-12. These two strains contain plasmids expressing *fdx_0135*, *fdr_0130* or *fdr_7100*, which proved that *fdx_0135* and *fdr_0130* were able to be deleted from the genome of strain H7029-14 and that *fdr_7100* was deleted from the genome of strain H7029-12 (Fig. 5a, b). In addition, three knock-out mutants (JH01, JH02 and JH03) were fermented to measure the proportion of epothilone A + B, but no obvious differences were seen compared with the respective control strains (Fig. 5c). This result was consistent with the results of RT-qPCR because the transcription levels of *fdx_0135*, *fdr_0130*, and *fdr_7100* increased significantly when these genes were overexpressed on plasmids. By comparing the loss rate of plasmid pEOK114 in strains JH01 and H7029-14, it was shown that after knocking out the gene *fdx_0135* from the chromosome, the plasmid was no longer lost (Additional file 1: Figure S9). These findings indicated that *fdx_0135* might be involved in the essential physiological functions of DSM 7029, and the other two genes *fdr_0130* and *fdr_7100* had the same speculation as *fdx_0135*. In summary, although these experiments did not confirm whether only these three genes are involved in the epoxidase activity of EpoK, our observations indicate that these three genes perform critical functions in DSM 7029.

## Discussion

Research progress in the pharmacology of epothilones has continuously promoted the development of epothilone synthetic techniques. In particular, the biosynthesis of epothilones in different heterologous hosts has been carried out since the epothilone biosynthetic gene cluster was first characterized and sequenced from *S. cellulosum*. Strain DSM 7029 has a fast growth cycle, and its ease of genetic manipulation shows enormous potential for high-yield epothilone production [18, 25]. However, the epoxidase EpoK, which is the final component encoded by the epothilone biosynthetic gene cluster, loses its normal activity in most heterologous systems due to the lack of high-efficiency redox partners. Although Kern and co-workers obtained an epothilone D conversion rate of 74.4% with the homologous redox partners Fdx8/FdR_B from *S. cellulosum* So ce56 in vitro [22], this pair of redox partners was not tested in vivo, and the high cost of enzyme purification and cofactors, low operational stability, and sensitivity of enzymes to reaction conditions were multiple factors limiting the use of this enzymatic conversion system in vitro for industrial applications [26]. Furthermore, there might be better redox partners to support the activity of EpoK to obtain a higher conversion rate. Inadvertently, we discovered that DSM 7029 expresses efficient redox partners to support EpoK. In this study, six ferredoxin genes and two ferredoxin reductase genes from DSM 7029 and the *fdx8*/*fdR_B* homologue *fdx_S4580*/*fdr_S2240* from *S. cellulosum* So0157-2 were investigated to find new efficient redox partners that support the catalytic activity of EpoK.

We found obvious effects of the high expression of the ferredoxin Fdx_0135 from DSM 7029 and the ferredoxin Fdx_S4580 from *S. cellulosum* So0157-2, both of which are hybrid [3Fe-4S]/[4Fe-4S] ferredoxins. In addition, the overexpression of *fdx_A6445* from the *Aquabacterium olei* strain NBRC 110486, which is highly homologous to *fdx_0135*, greatly increased the proportion of epothilone A + B. Previous studies revealed that redox partner-mediated electron transfer is often the rate-limiting event in a P450 catalytic cycle and that ferredoxin is a key protein that directly interacts with P450 [27]. Typical surrogate redox partners, such as *Pseudomonas putida* putidaredoxin (Pdx)/putidaredoxin reductase (PdR), spinach ferredoxin/ferredoxin reductase, and bovine adrenodoxin (Adx)/adrenodoxin reductase (AdR), are not good redox partners for EpoK; all of these are [2Fe-2S] ferredoxins [22]. Moreover, the [2Fe-2S] ferredoxins Fdx_2170, Fdx_4560, and Fdx_6105 and the [4Fe-4S] dicluster ferredoxins Fdx_2730 and Fdx_5185 were unsuccessful in this study. Altogether, these results indicated that EpoK might preferentially interact with hybrid [3Fe-4S]/[4Fe-4S] ferredoxins. These ferredoxins have strong electrostatic interactions between the negatively charged amino acids on the iron–sulfur side and the positively charged residues located on the EpoK proximal side. In addition, the distance between the iron–sulfur cluster and the haeme iron is appropriate.

The other influencing protein, ferredoxin reductase, accepts a hydride from NAD(P)H, in turn reducing two equivalents of ferredoxin; these proteins exhibit a similar interaction with P450/ferredoxin [27]. In this study, by the whole-cell biotransformation of epothilone C, we identified three ferredoxin reductases that support the catalytic activity of EpoK. FdR_0130 and FdR_7100 delivered electrons to Fdx_0135, but FdR_S2240 could not deliver electrons to the ferredoxins from DSM 7029 and interacted with only Fdx_S4580 to support the activity of EpoK.

Initially, we sought to confirm whether only Fdx_0135, FdR_0130, and FdR_7100 support the catalytic activity of EpoK by knocking out their genes in DSM 7029. However, these three redox genes could not be deleted without plasmid-mediated complementation in H7029-14 and H7029-12. Ferredoxins and ferredoxin reductases are electron carriers that shuttle electrons, which are linked to biochemical pathways for energy transduction, nutrient assimilation, and primary metabolism in organisms [28]. We found that *fdr_0130* is adjacent to *fdx_0135* in the bacterial strain DSM 7029 and that these two genes are located in the same operon as some genes related to sulfur metabolism. The sulfur metabolism pathways of strain DSM 7029 predicted by KEGG revealed that four genes in this operon are involved in sulfate reduction and assimilation (Additional file 1: Figure S10). Therefore, we concluded that Fdx_0135 and FdR_0130 may constitute a pair of electron transfer partners that deliver electrons to sulfite reductase and that FdR_7100 may be a prevalent redox protein supporting the physiological function of DSM 7029 [29].

## Conclusion

In conclusion, our results demonstrated that the redox pairs Fdx_0135/FdR_0130 and Fdx_0135/FdR_7100 from DSM 7029 efficiently supported the catalytic activity of EpoK with a potential approaching that of the redox pair Fdx_S4580/FdR_S2240 from *S. cellulosum* So0157-2. In addition, the overexpression of redox protein genes was a simple and effective approach to screen for electron transfer partners to support the P450 EpoK. This strategy could be used to screen and identify the electron transport partners supporting other P450 enzymes. The engineered DSM 7029 strain had a high epothilone epoxidation conversion rate, providing substantial potential for future in vivo studies of P450s and industrial biosynthesis of epoxy-epothilones.

## Materials and methods

### Materials

High-fidelity DNA polymerase was purchased from TSINGKE Biological Technology (Beijing, China), a One-step PCR Cloning Kit was purchased from Novoprotein Scientific (Shanghai, China), restriction endonucleases were purchased from Thermo Fisher Scientific (Massachusetts, United States), and the kits used for plasmid and RNA extraction were obtained from Generay Biotech (Shanghai, China). Other chemicals were purchased as follows: epothilone standard samples, Meilun Biotechnology (Dalian, China); methanol (MACLIN, China), acetonitrile (Sinopharm Chemical, China) and Amberlite™ XAD-16 resin (Mosu, Shanghai, China). The primers used in this study were synthesized by TSINGKE Biological Technology and Generay Biotech, and the genes were sequenced by TSINGKE Biological Technology and GENEWIZ (Suzhou, China).

### Bacterial strains and culture conditions

All DSM 7029 mutants used in this study are listed in Table 3. *E. coli* DH10B was employed for cloning and was cultured in LB medium. *Schlegelella brevitalea* DSM 7029 mutants were cultured on CYMG medium as previously described by Lei et al. [24] at 30 °C, and 1.5% agar was added to the solid medium. Suitable precursor compounds were added to CYMG liquid medium for fermentation as previously described by Bian et al. [18], except the concentration of methylmalonic acid was changed to 200 mg L^−1^ and 2% XAD-16 resin was used. Fermentation was conducted at 30 °C and 200 rpm for 6 days. Whole-cell biotransformation was conducted in 50 mL of CYMG medium. Appropriate concentrations of antibiotics were used to maintain the plasmids: 100 μg mL^−1^ ampicillin, 50 μg mL^−1^ kanamycin, 5 μg mL^−1^ gentamicin, 50 μg mL^−1^ apramycin, 150 μg mL^−1^ hygromycin B for DSM 7029, and 200 μg mL^−1^ hygromycin B for DH10B.

### Plasmids and strains construction

The plasmids used in this study are described in Additional file 1: Table S2, and the primers used in this study are listed in Additional file 1: Table S3. All six ferredoxin genes and two ferredoxin reductase genes were cloned from the genome of DSM 7029 by PCR. *fdx_A6445* was amplified from the synthetic plasmid pEOK202. Then, these genes were ligated to P15A-Amp^r^-P_*kan*_ (vector 1, PCR amplified from pEOK001) using In-Fusion Cloning products to obtain pEOK002 to pEOK011. The *Tn5*-*kan* promoter was fused to all ferredoxin or ferredoxin reductase genes. Then, to express *epoK* and overexpress ferredoxin or ferredoxin reductase genes, oriV-trfA derivate-Apra^r^ (vector 2) was cloned by PCR from pEOK117 and ligated to all P_*kan*_-*fdx/fdr* constructs, including the synthetic fragments *P*_*kan*_-*fdx_S4580*, *P*_*kan*_-*fdr_S2240*, and *P*_*kan*_-*fdx_S4580*-*P*_*kan*_-*fdr_S2240*, by In-Fusion Cloning or BioBrick restriction digestion and ligation; in this manner, pEOK101 to pEOK116 were successfully constructed. These resulting plasmids were verified by resistance selection, restriction digestion analysis, and sequencing. pEOK102 to pEOK116 were transformed into the epothilone C/D high-yield strain H7029-1 to obtain strains H7029-2 to H7029-116, sequentially. To establish the DSM 7029 whole-cell biotransformation systems, ten plasmids (pEOK101, pEOK102, pEOK103, pEOK109, and pEOK111 to pEOK116) were transformed into the wild-type strain DSM 7029 to obtain strains 7029-1 to 7029-10, sequentially. Test primers specific for the functional genes were used to screen for the correct single colony.

### Fermentation, extraction, and analysis of epothilones

After fermentation in a 500 mL volume for 6 days, standard sieves (100 mesh) were used to collect the XAD-16 resins and were washed several times using deionized water. Then, after drying at 30 °C, the resins were incubated with 100 mL of ethyl acetate at 30 °C and 200 rpm for 24 h. The extract liquor was filtered through a 0.22 μm organic filter membrane, centrifuged at 13,000 rpm for 10 min, diluted 5- or 10-fold, and analysed by HPLC. HPLC was performed in a 1260 Infinity system (Agilent Technologies, Santa Clara, USA) with an Ultimate XBC18 column (4.6 × 250 mm, 5-μm particle diameter; Welch, Shanghai, China). Test samples were separated at a flow rate of 1 mL min^−1^ and a temperature of 30 °C. The injection volume was set at 10 μL each time, and the sample was monitored at 249 nm. After equilibration of the column with 35% solution A (ultrapure water with 0.2% acetic acid) and 65% solution C (methanol), the samples were separated on a gradient starting at 65% solution C for 5 min, increasing to 75% at 15 min, increasing to 80% at 25 min, and ending at 40 min. Standard samples of 2.5 mg L^−1^, 5 mg L^−1^, 7.5 mg L^−1^, 12.5 mg L^−1^, 15 mg L^−1^, 20 mg L^−1^, and 25 mg L^−1^ were also analysed to generate the standard curve (Additional file 1: Figure S11).

### Whole-cell biotransformation of epothilone C in the engineered DSM 7029 strains

We used a rotary evaporator to prepare a large amount of epothilone C crude product, which was dissolved in DMSO (Additional file 1: Figure S6). In this study, we measured the whole-cell conversion of epothilone C to epothilone A. Strains 7029-1 to 7029-10 were cultured in 50 mL of CYMG for 24 h, and the mouth of the culture bottle was sealed with gauze to attain a higher O_2_ concentration (OD_600_ = 6.0 ± 1.0). At this time point, epothilone C was added to the 50-mL conversion system to a concentration of 40 mg L^−1^. After continued culture for different durations (6 h, 12 h, 18 h, 24 h, and 30 h), an equal volume of ethyl acetate was added to extract fermentation products at 30 °C and 200 rpm for 24 h. After extraction, the samples were shaken again, and the supernatant was removed immediately and centrifuged at 13,000 rpm for 10 min. The supernatant was then filtered for HPLC analysis.

### Gene overexpression analysis by RT-qPCR

DSM 7029 mutant cells were harvested at an OD_600_ value of approximately 4.3 ± 0.5, at approximately 18 h. RNA extraction was carried out with an RNAprep Pure Cell/Bacteria Kit (TIANGEN, China) according to the manufacturer’s instructions. Genomic DNA was removed by using DNA Eraser supplied in the 1st Strand cDNA Synthesis Kit (Takara), and its removal was confirmed by PCR. cDNA was synthesized using HiScript^R^ III RT SuperMix for qPCR (+ gDNA wiper) (Vazyme Biotech, China) for RT-PCR with 800 ng of total RNA. Gene expression was analysed using real-time RT-PCR with ChamQ™ Universal SYBR^R^ qPCR Master Mix (Vazyme Biotech, China). PCR was conducted in a BIO-RAD Real-Time System with the following thermal cycling program: 30 s at 95 °C, followed by 40 cycles of 10 s at 95 °C and 30 s at 60 °C. The *mraW* gene was used as the normalization signal. The amplification primers used for each gene are listed in Additional file 1: Table S3. The transcription levels of *fdx_0135*, *fdr_0130*, and *fdr_7100* were measured.

## Supplementary information

Additional file 1: Figure S1. Alignment of the tertiary structures of P450cam and EpoK. Figure S2. Visual representation of the location of the six selected ferredoxin genes and two selected ferredoxin reductase genes. Figure S3. Multiple sequence alignment of EpoK from three sources. Figure S4. Results of colony PCR to verify the transformation of plasmids pEOK102 to pEOK116 into H7029-1. Table S1. Yields and component proportion of epothilones in mutants of strain H7029-1. Figure S5. Comparison of the protein sequence and structural alignment between Fdx_0135 and Fdx_A6445. Figure S6. HPLC results of epothilone C crude extracts. Figure S7. Results of colony PCR to verify the transformation of the corresponding plasmids into the wild-type strain DSM 7029. Figure S8. Verification of *fdx_0135* knock-out by two-step homologous recombination in strain H7029-1. Figure S9. Comparison of the loss rate of plasmid pEOK114 (oriV-trfA-Apra^r^-P_*kan*_-*epoK*-P_*kan*_-*fdx_0135*-P_*kan*_-*fdr_0130*) in strains JH01 and H7029-14. Figure S10. Sulfur metabolism network in strain DSM 7029, as predicted by KEGG. Table S2. List of plasmids used in this study. Table S3. List of primers used in this study. Figure S11. Epothilone standard sample curves calculated by the peak areas at different concentrations (2.5 mg L^−1^, 5 mg L^−1^, 7.5 mg L^−1^, 12.5 mg L^−1^, 15 mg L^−1^, 20 mg L^−1^, and 25 mg L^−1^).

## Data Availability

The dataset(s) supporting the conclusions of this article is (are) included within the article [and its additional file(s)].
