# Supplementary material for: Heterologous redox partners supporting the efficient catalysis of epothilone B biosynthesis by EpoK in Schlegelella brevitalea
Source: Microb Cell Fact. 2020 Sep 15;19:180. doi: 10.1186/s12934-020-01439-5 (PMC7493146; doi:10.1186/s12934-020-01439-5)
Supplement: Supplementary file 1 — Additional file 1: Figure S1. Alignment of the tertiary structures of P450cam and EpoK. Figure S2. Visual representation of the location of the six selected ferredoxin genes and two selected ferredoxin reductase genes. Figure S3. Multiple sequence alignment of EpoK from three sources. Figure S4. Results of colony PCR to verify the transformation of plasmids pEOK102 to pEOK116 into H7029-1. Table S1. Yields and component proportion of epothilones in mutants of strain H7029-1. Figure S5. Comparison of the protein sequence and structural alignment between Fdx_0135 and Fdx_A6445. Figure S6. HPLC results of epothilone C crude extracts. Figure S7. Results of colony PCR to verify the transformation of the corresponding plasmids into the wild-type strain DSM 7029. Figure S8. Verification of fdx_0135 knock-out by two-step homologous recombination in strain H7029-1. Figure S9. Comparison of the loss rate of plasmid pEOK114 (oriV-trfA-Aprar-Pkan-epoK-Pkan-fdx_0135-Pkan-fdr_0130) in strains JH01 and H7029-14. Figure S10. Sulfur metabolism network in strain DSM 7029, as predicted by KEGG. Table S2. List of plasmids used in this study. Table S3. List of primers used in this study. Figure S11. Epothilone standard sample curves calculated by the peak areas at different concentrations (2.5 mg L−1, 5 mg L−1, 7.5 mg L−1, 12.5 mg L−1, 15 mg L−1, 20 mg L−1, and 25 mg L−1). [file 12934_2020_1439_MOESM1_ESM.docx]

**Heterologous redox partners supporting the efficient catalysis of epothilone B biosynthesis by EpoK in** ***Schlegelella brevitalea***

[Junheng Liang]^1#^ [Huimin Wang]^1#^ [Xiaoying Bian]^2^ [Youming Zhang]^2^ [Guoping Zhao]^1, 3^ [Xiaoming Ding]^1*^

^1^[Collaborative Innovation Center for Genetics and Development, State Key Laboratory of Genetic Engineering, Department of Microbiology, School of Life Sciences, Fudan University, Shanghai, People’s Republic of China]

^2^[Shandong University-Helmholtz Institute of Biotechnology, State Key Laboratory of Microbial Technology, School of Life Sciences, Shandong University, Qingdao, Shandong, People’s Republic of China]

^3^[CAS Key Laboratory of Synthetic Biology, Institute of Plant Physiology and Ecology, Shanghai Institutes for Biological Sciences, Chinese Academy of Sciences, Shanghai, People’s Republic of China]

# Junheng Liang and Huimin Wang contributed equally to this work.

*** Correspondence:**

Xiaoming Ding: Department of Microbiology, School of Life Sciences, Fudan University, Shanghai, 200438, People’s Republic of China.

**E-mail:** [xmding74@fudan.edu.cn]


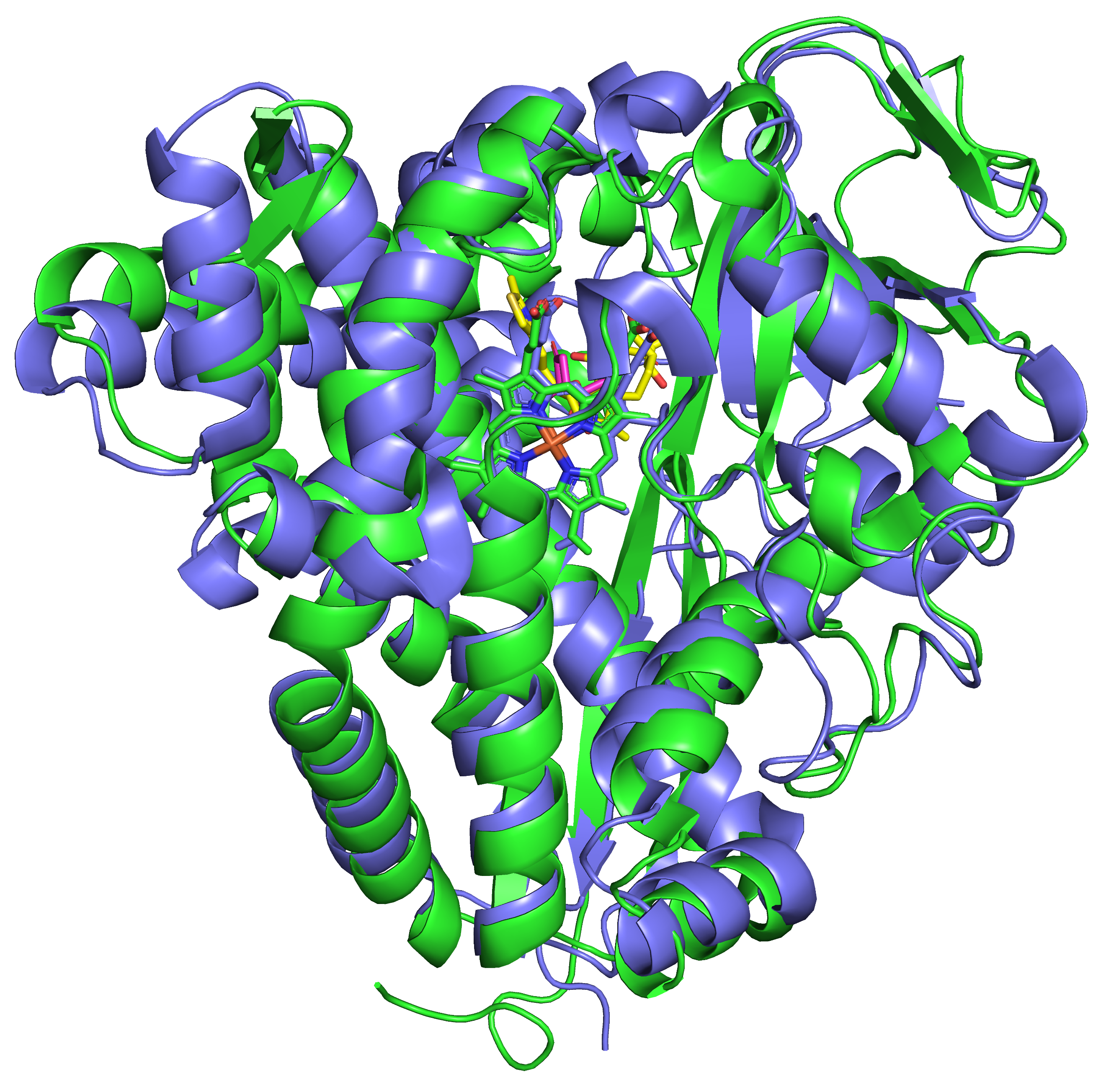


**Figure S1.** **Alignment of the tertiary structures of P450cam and EpoK.** P450cam is shown in green, PDB ID: 2H7Q; EpoK is shown in blue, PDB ID: 1Q5E. These structures exhibited the typical triangular prism shape.


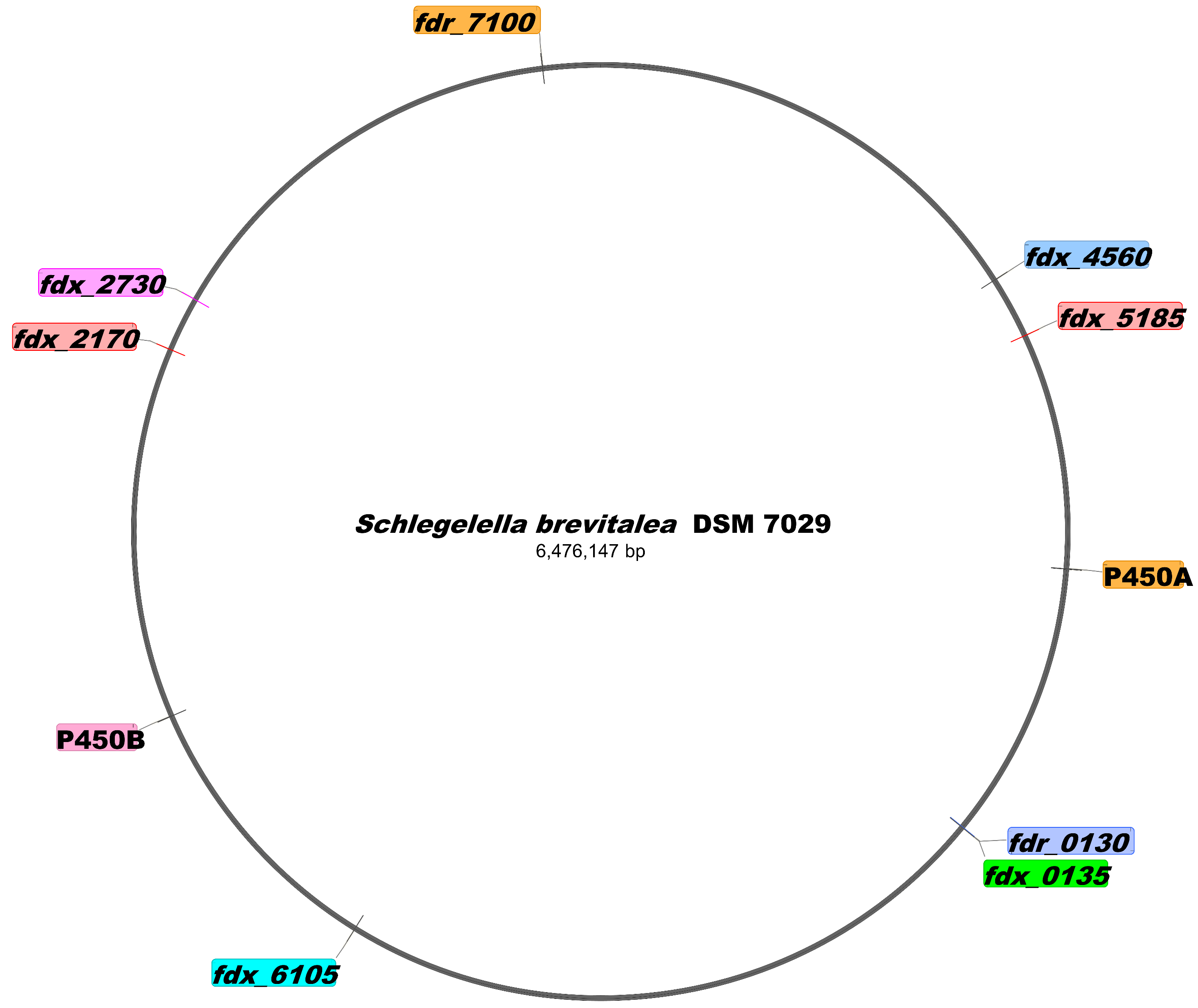


**Figure S2. Visual representation of the location of the six selected ferredoxin genes and two selected ferredoxin reductase genes.** The sequences of two cytochrome P450 enzymes, P450A (*AAW51_RS07440*) and P450B (*AAW51_RS18535*), were added. The genomic locations of the depicted genes were retrieved from the National Center for Biotechnology Information (NCBI).


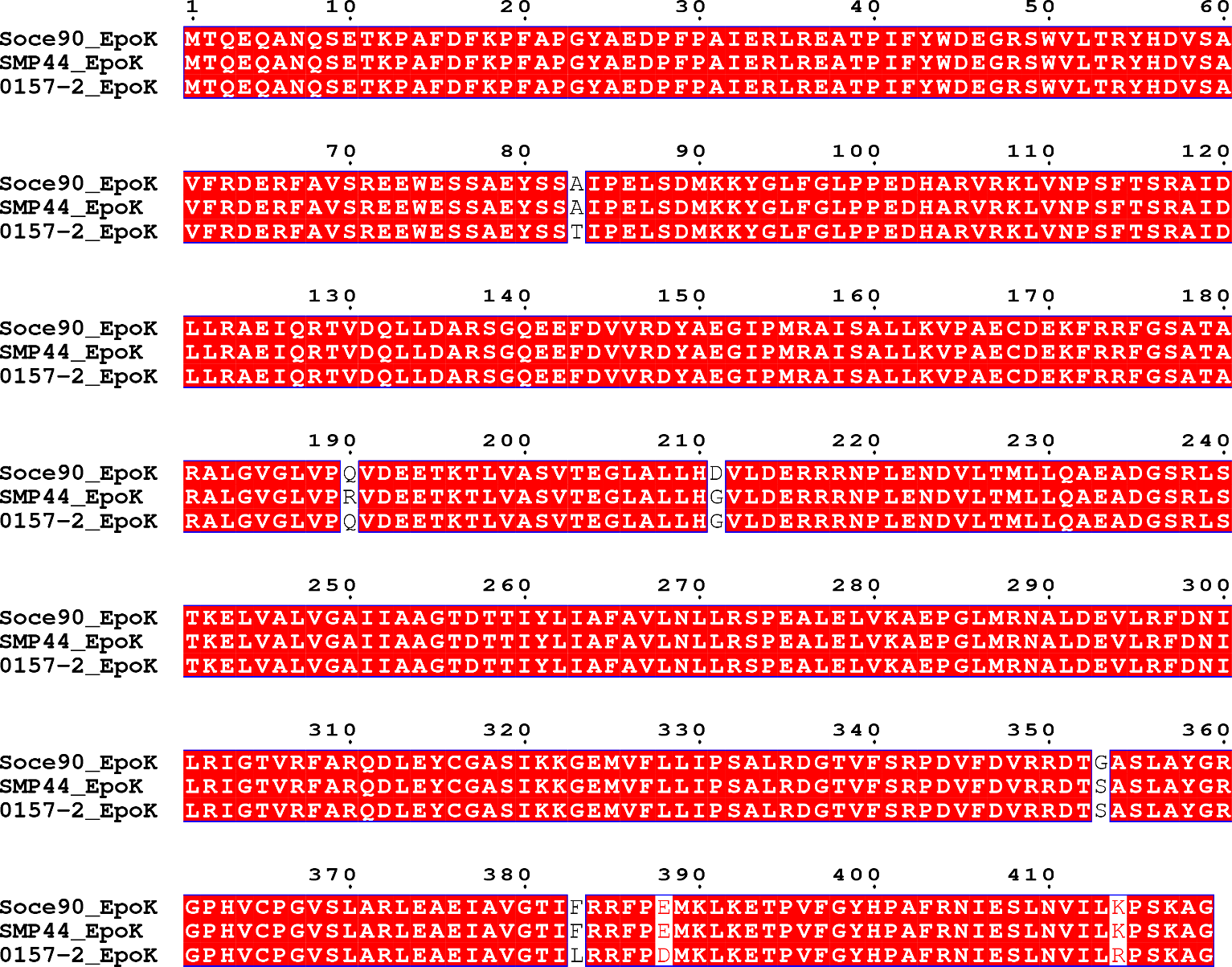


**Figure S3. Multiple sequence alignment of EpoK from three sources.** The amino acid sequences were aligned using ClustalW (<https://www.genome.jp/tools-bin/clustalw>). The alignment was visualized using ESPript 3.0 (http://espript.ibc p.fr/ESPript/cgi-bin/ESPript.cgi).


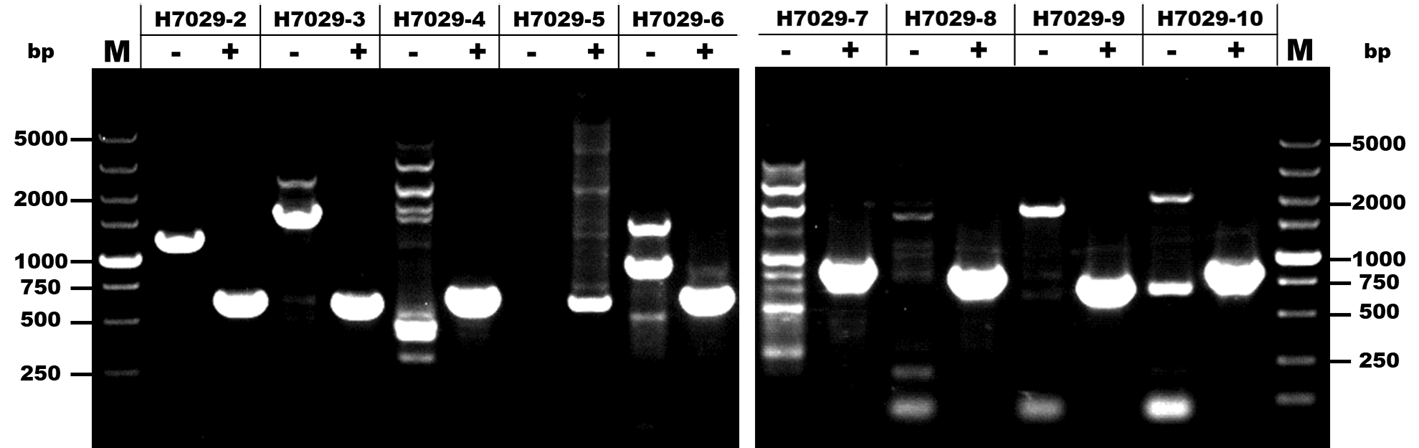

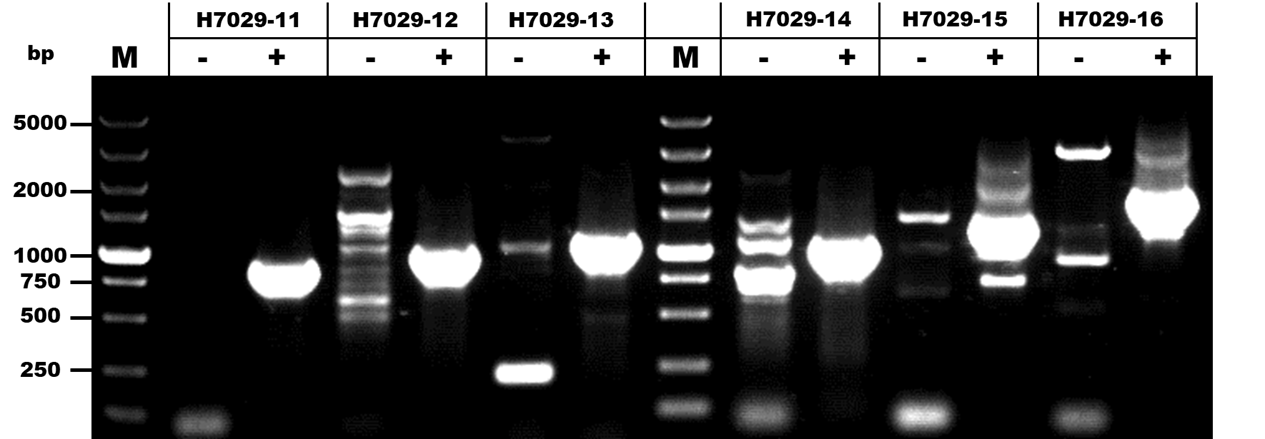


**Figure S4.** **Results of colony PCR to verify the transformation of plasmids pEOK102 to pEOK116 into H7029-1.** When these plasmids were successfully transformed into H7029-1, the lengths of the PCR-amplified fragments were 655 bp, 629 bp, 686 bp, 669 bp, 683 bp, 853 bp, 790 bp, 711 bp, 859 bp, 845 bp, 969 bp, 1080 bp, 1029 bp, 1258 bp, and 1679 bp in H7029-2 to H7029-16, sequentially. In the control group, the genome of H7029-1 was used as the template, and the correct fragment could not be amplified by PCR.

**Table S1 Yields and component proportion of epothilones in mutants of strain H7029-1**

| **H7029 mutants** | **Epothilone A+B**  **(mg L^–1^)** | **Total Epothilone**  **(mg L^–1^)** | **A+B proportion (%)** |
| --- | --- | --- | --- |
| H7029-1 | 0.000 ± 0.000 | 45.867 ± 0.575 | 0.00 ± 0.00 |
| H7029-2 | 33.857 ± 4.636 | 59.351 ± 4.817 | 56.87 ± 3.23 |
| H7029-3 | 47.633 ± 4.121 | 54.738 ± 2.433 | 86.91 ± 3.62 |
| H7029-4 | 21.452 ± 0.544 | 55.345 ± 4.135 | 38.86 ± 1.98 |
| H7029-5 | 36.819 ± 3.694 | 56.199 ± 2.984 | 65.41 ± 3.07 |
| H7029-6 | 32.858 ± 7.015 | 60.071 ± 5.531 | 54.27 ± 7.06 |
| H7029-7 | 33.498 ± 0.968 | 53.916 ± 2.599 | 62.24 ± 3.73 |
| H7029-8 | 35.631 ± 4.928 | 57.601 ± 5.453 | 61.68 ± 2.89 |
| H7029-9 | 43.518 ± 5.261 | 55.659 ± 5.172 | 78.05 ± 2.52 |
| H7029-10 | 41.340 ± 4.129 | 51.583 ± 2.806 | 80.02 ± 3.94 |
| H7029-11 | 41.087 ± 1.347 | 57.297 ± 1.533 | 71.71 ± 0.79 |
| H7029-12 | 37.455 ± 7.707 | 57.868 ± 7.557 | 64.33 ± 4.69 |
| H7029-13 | 39.234 ± 2.306 | 60.823 ± 4.023 | 64.66 ± 5.18 |
| H7029-14 | 50.202 ± 3.391 | 55.792 ± 4.081 | 90.00 ± 0.54 |
| H7029-15 | 51.626 ± 5.171 | 62.950 ± 4.913 | 81.92 ± 1.92 |
| H7029-16 | 48.913 ± 6.315 | 55.622 ± 5.500 | 87.75 ± 2.94 |


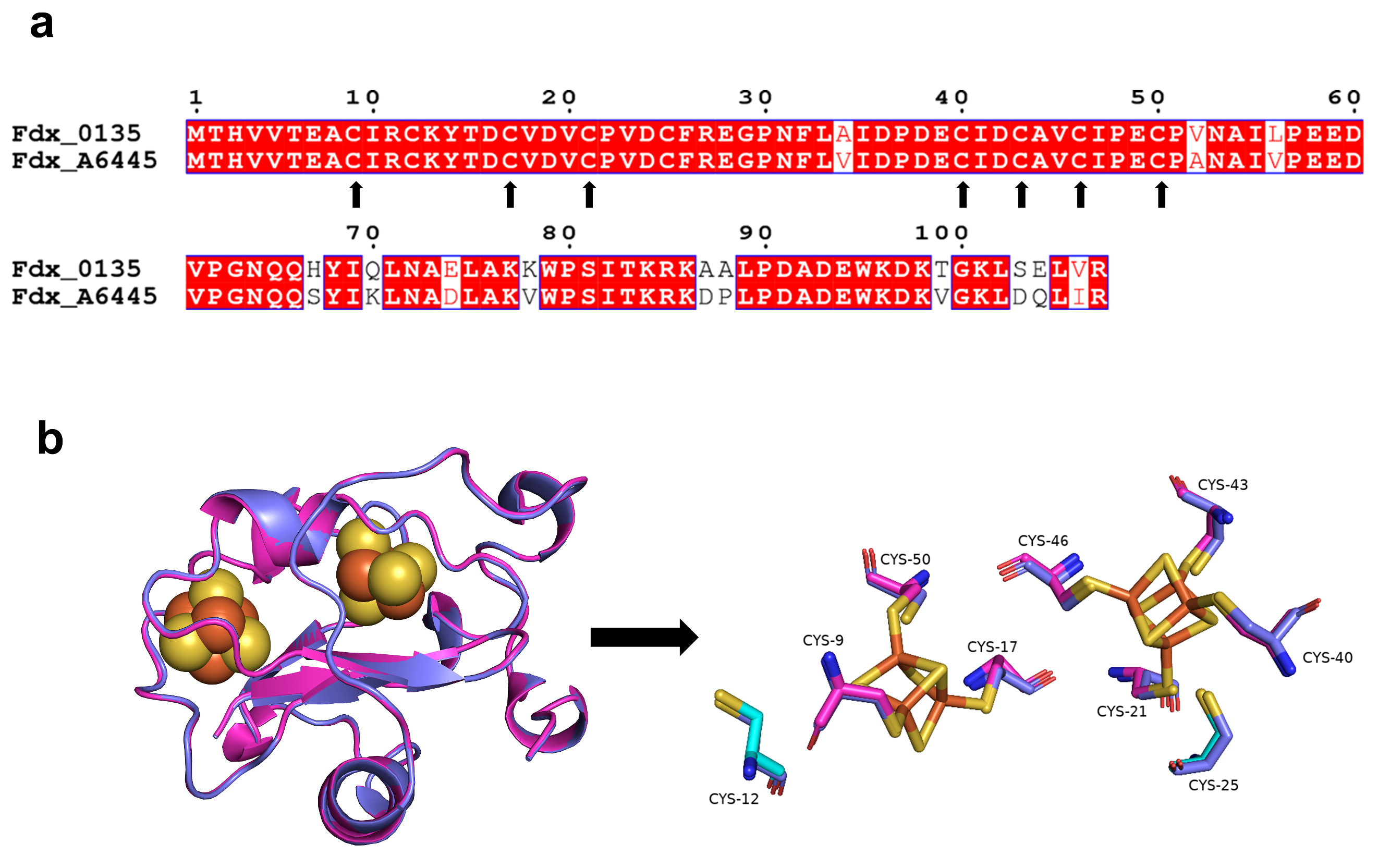


**Figure S5. Comparison of the protein sequence and structural alignment between Fdx_0135 and Fdx_A6445.** a. Amino acid sequence alignment of the two ferredoxins. The cysteine residues bound to the iron-sulfur clusters are indicated by black arrows. b. The structural alignment of the two ferredoxins was visualized using PyMOL 2.3.3, and the tertiary structures of the two proteins were predicted by Swiss-Model (https://swissmodel. expasy.org/). The cysteine residues C9, C17, C21, C25, C40, C43, C46, C50 are bound to two iron-sulfur clusters. Fdx_0135 is shown in magenta, Fdx_A6445 is shown in purple, iron atoms are shown in orange, and sulfur atoms are shown in yellow.


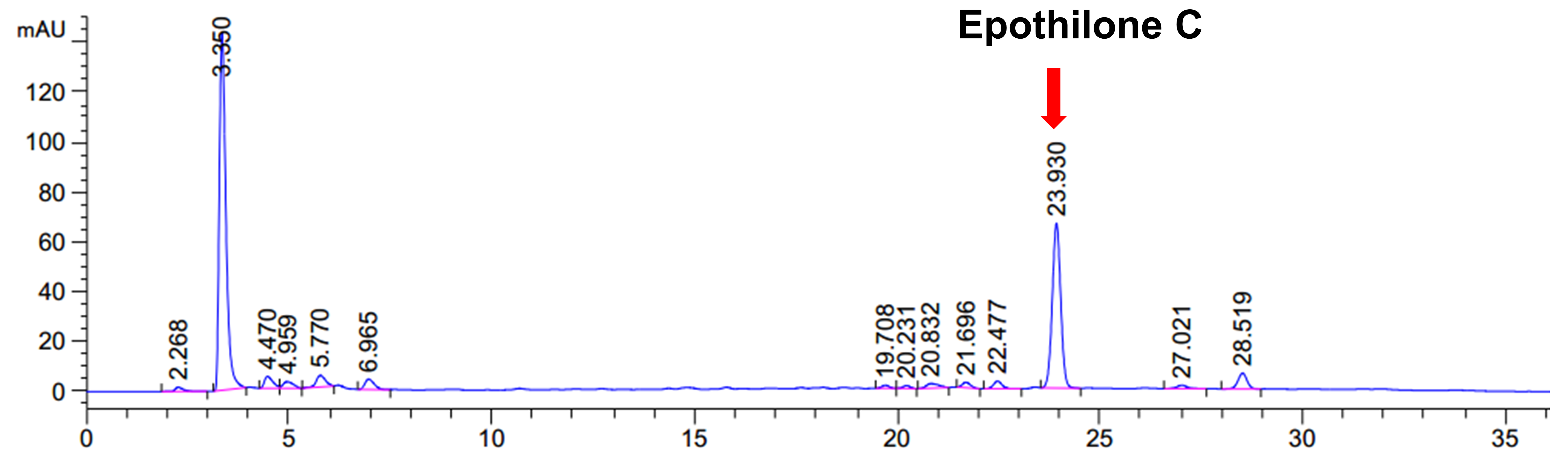


**Figure S6. HPLC results of epothilone C crude extracts.** Epothilone C crude extracts were concentrated and dissolved in DMSO, and the tested sample was diluted 200-fold.


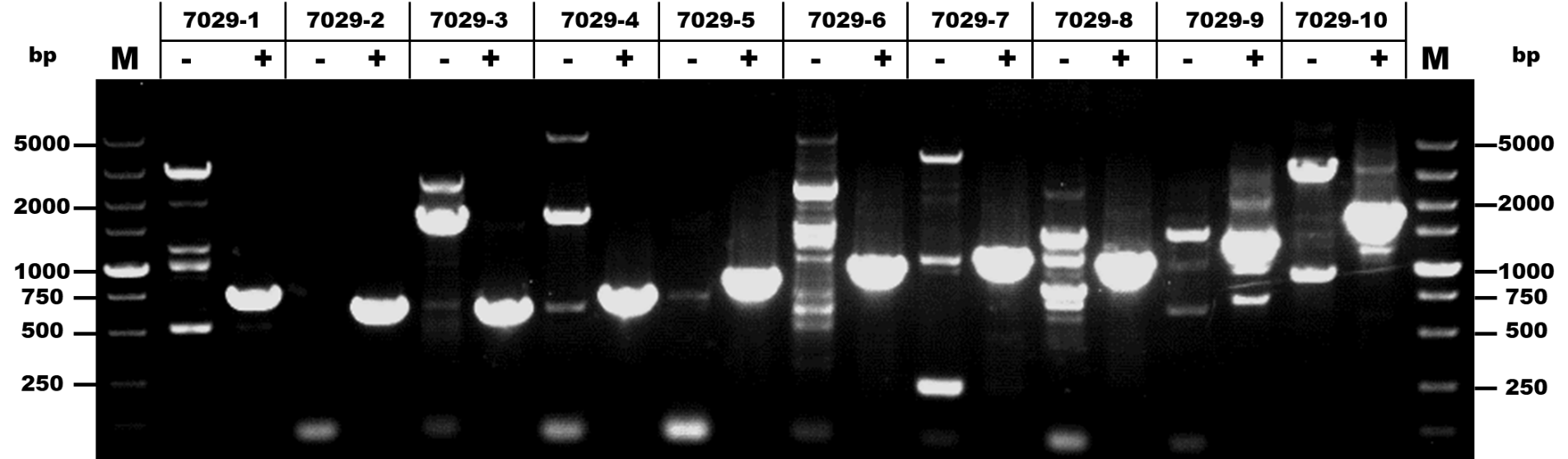


**Figure S7. Results of colony PCR to verify the transformation of the corresponding plasmids into the wild-type strain DSM 7029.** When these plasmids were successfully transformed into DSM 7029, the lengths of the PCR-amplified fragments were 742 bp, 655 bp, 629 bp, 711 bp, 845 bp, 969 bp, 1080 bp, 1029 bp, 1258 bp, and 1679 bp in 7029-1 to 7029-10, sequentially. In the control group, the genome of DSM 7029 was used as the template, and the correct fragment could not be amplified by PCR.


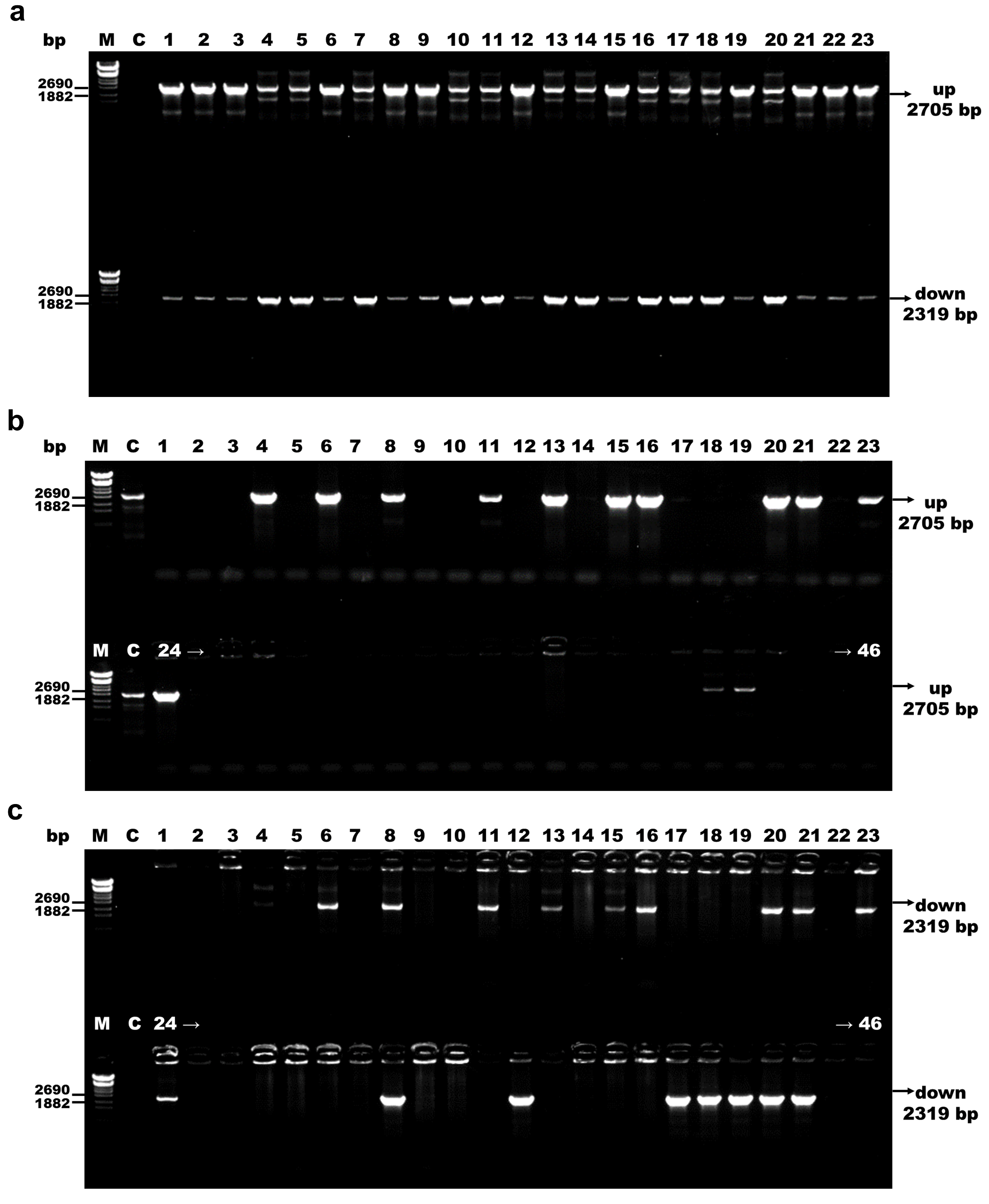


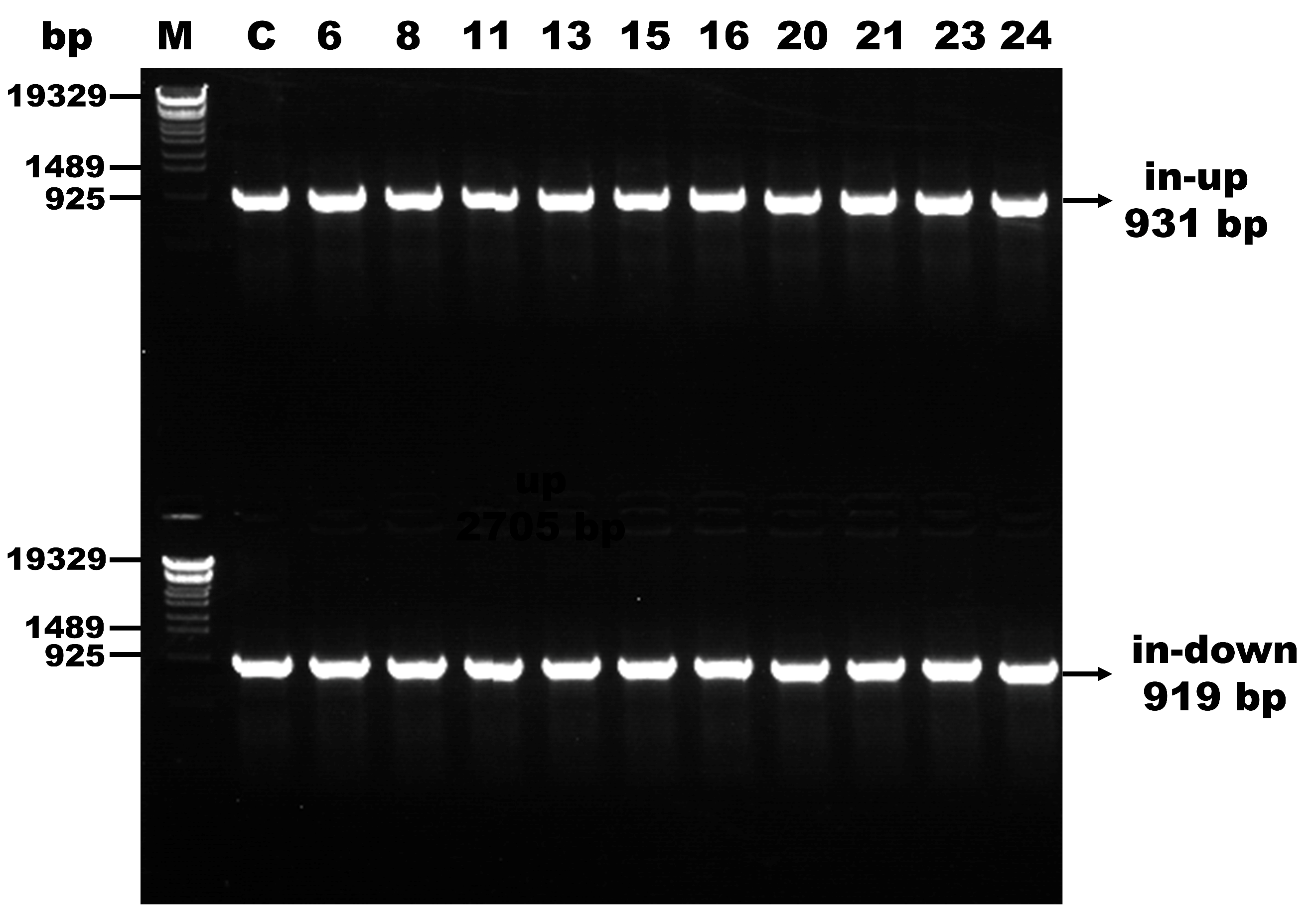


**d**

**Figure S8. Verification of *fdx_0135* knock-out by two-step homologous recombination in strain H7029-1.**

1. PCR analysis of single-crossover homologous recombination of *fdx_0135* selected by hygromycin resistance without the addition of sucrose for negative screening. The lengths of the upstream and downstream fragments flanking the upstream and downstream homologous arms of the knock-out gene were 2705 bp and 2319 bp, respectively. Although the upstream and downstream fragments of some colonies were rarely amplified, further internal PCR verification indicated that *fdx_0135* was not deleted. We confirmed that all colonies were single-crossover mutants. Subsequently, we selected colony 1 for the second step of homologous recombination.
2. PCR analysis of the upstream fragments flanking the homologous arm after the second step of selection of double-crossover mutants with sucrose (non-hygromycin added).
3. PCR analysis of the downstream fragments flanking the homologous arm after the second step of selection of double-crossover mutants with sucrose (non-hygromycin added).
4. Internal verification of the ten colonies with suspected double-crossover. The results of the second step of homologous recombination showed that twenty-eight colonies were restored to the wild-type state, eight colonies remained in the single-crossover state, and ten colonies had suspected double-crossover. However, further internal verification of the ten colonies demonstrated that all were false-positive colonies.


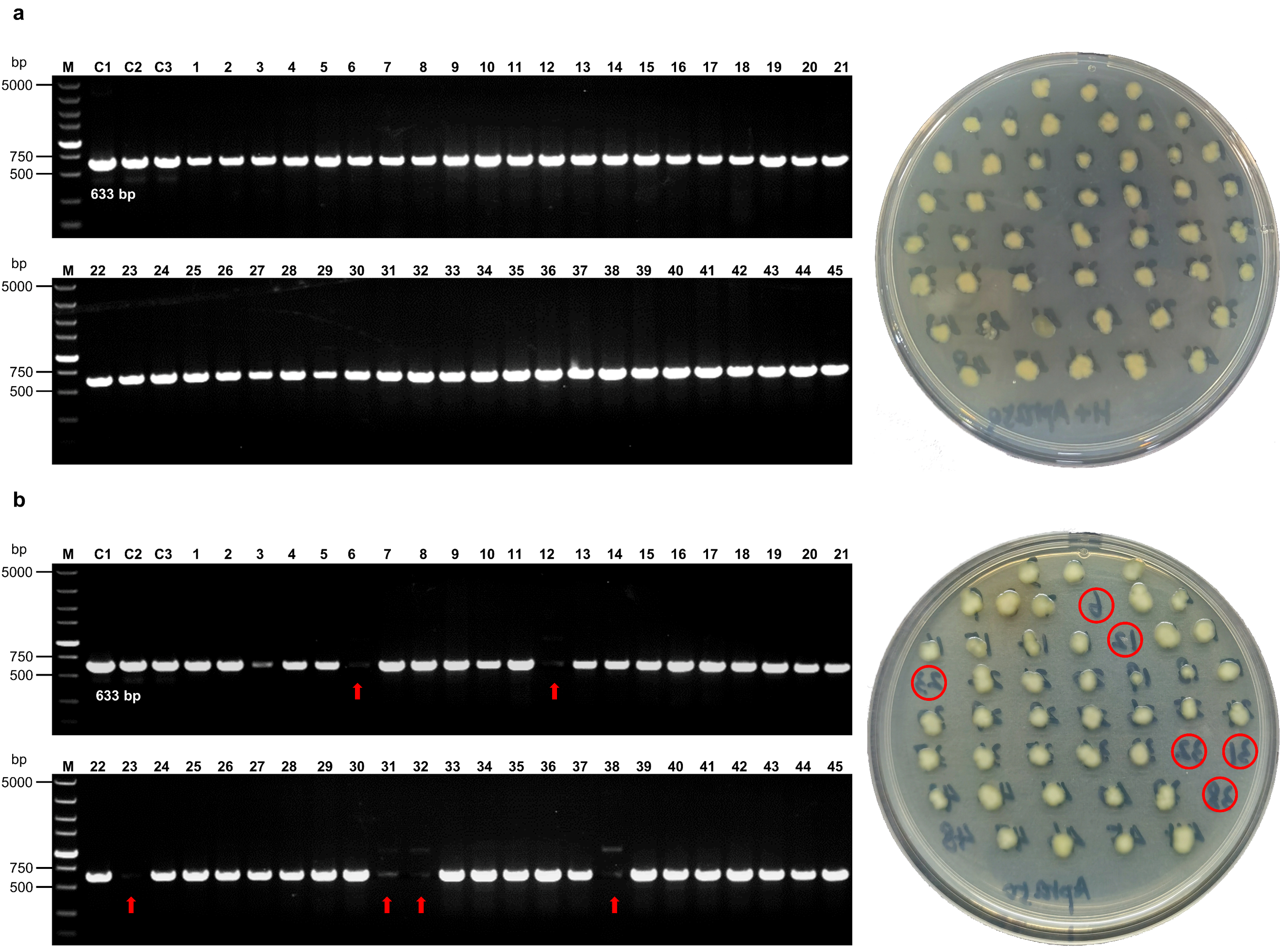


**Figure S9 Comparison of the loss rate of plasmid pEOK114 (oriV-trfA-Apra^r^-P*_kan_*-*epoK*­-P*_kan_*-*fdx_0135-*P*_kan_-fdr_0130*) in strains JH01 and H7029-14.** To verify that Fdx_0135 is necessary for DSM 7029 growth, we designed an experiment to assess the loss of plasmid pEOK114 in strains JH01 and H7029-14. These two strains were transferred to culture in CYMG medium without apramycin for 8 rounds of culture. A single colony was then isolated on a CYMG solid agar plate without apramycin, and 45 single colonies were selected for PCR and resistance analysis. a. The loss rate of pEOK114 in strain JH01, as determined by PCR analysis and resistance analysis. The loss rate of pEOK114 was 0%. b. The loss rate of pEOK114 in strain H7029-14, as determined by PCR and resistance analysis. The loss rate of pEOK114 was 13.33%. Colonies 6, 12, 23, 31, 32, and 38, with pEOK114 loss, are marked with red arrows and circles.

**
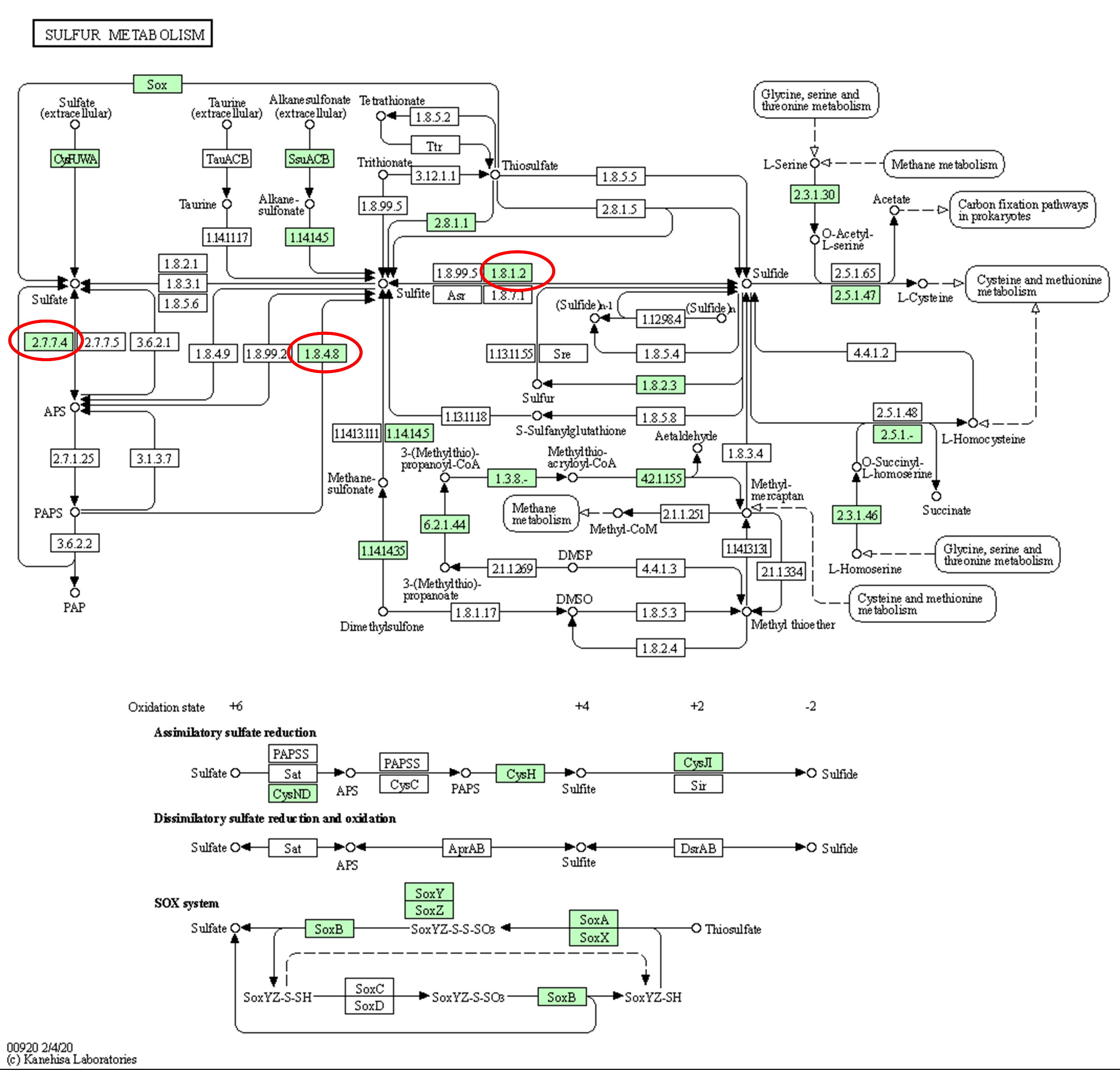
**

**Figure S10. Sulfur metabolism network in strain DSM 7029, as predicted by KEGG.** **EC: 2.7.7.4** (*AAW51_RS10145*: *cysN*, sulfate adenylyltransferase subunit 1; *AAW51_RS10150*: *cysD*; sulfate adenylyltransferase subunit 2); **EC: 1.8.4.8** (*AAW51_RS10155*: *cysH*, phosphoadenosine phosphosulfate reductase); **EC: 1.8.1.2** (*AAW51_RS10165*: *cysI*, sulfite reductase (NADPH) hemoprotein beta-component).

The enzymes corresponding to the four genes in the operons of *fdx_0130* and *fdr_0130* are marked with red ellipses in the sulfur metabolism network.

**Table S2** List of plasmids used in this study

| **Plasmid** | **Relevant genotype/property** | **Source** |
| --- | --- | --- |
| pEOK001 | p15A ori, Amp^­r^, *epoK* with the P*_kan_* promoter | Laboratory stock |
| pEOK002 | p15A ori, Amp^r^, *fdx_0135* with the P*_kan_* promoter, with *EcoR*I, *Xba*I, *Spe*I and *Pci*I restriction sites | This study |
| pEOK003 | p15A ori, Amp^r^, *fdx_2170* with the P*_kan_* promoter | This study |
| pEOK004 | p15A ori, Ampr, *fdx_2730* with the P*_kan_* promoter, with *EcoR*I, *Xba*I, *Spe*I and *Pci*I restriction sites | This study |
| pEOK005 | p15A ori, Amp^r^, *fdx_4560* with the P*_kan_* promoter | This study |
| pEOK006 | p15A ori, Amp^r^, *fdx_5185* with the P*_kan_* promoter | This study |
| pEOK007 | p15A ori, Ampr, *fdx_6105* with the P*_kan_* promoter, with *EcoR*I, *Xba*I, *Spe*I and *Pci*I restriction sites | This study |
| pEOK008 | p15A ori, Amp^r^, *fdx_A6445* with the P*_kan_* promoter | This study |
| pEOK009 | p15A ori, Amp^r^, *fdr_0130* with the P*_kan_* promoter | This study |
| pEOK010 | p15A ori, Amp^r^, *fdr_7100* with the P*_kan_* promoter | This study |
| pEOK011 | p15A ori, Amp^r^, *fdx_0135-fdr_0130* with the P*_kan_* promoter | This study |
| pEOK101 | oriV-trfA derivate, Apra^r^, empty vector | This study |
| pEOK102 | oriV-trfA derivate, Apra^r^, *epoK* with the P*_kan_* promoter, with *EcoR*I, *Xba*I, *Spe*I and *Pci*I restriction sites | This study |
| pEOK103 | oriV-trfA derivate, Apra^r^, *epoK and fdx_0135* with the P*_kan_* promoter | This study |
| pEOK104 | oriV-trfA derivate, Apra^r^, *epoK and fdx_2170* with the P*_kan_* promoter | This study |
| pEOK105 | oriV-trfA derivate, Apra^r^, *epoK and fdx_2730* with the P*_kan_* promoter | This study |
| pEOK106 | oriV-trfA derivate, Apra^r^, *epoK and fdx_4560* with the P*_kan_* promoter | This study |
| pEOK107 | oriV-trfA derivate, Apra^r^, *epoK and fdx_5185* with the P*_kan_* promoter | This study |
| pEOK108 | oriV-trfA derivate, Apra^r^, *epoK and fdx_6105* with the P*_kan_* promoter | This study |
| pEOK109 | oriV-trfA derivate, Apra^r^, *epoK and fdx_S4580* with the P*_kan_* promoter | This study |
| pEOK110 | oriV-trfA derivate, Apra^r^, *epoK and fdx_A6445* with the P*_kan_* promoter | This study |
| pEOK111 | oriV-trfA derivate, Apra^r^, *epoK and fdr_0130* with the P*_kan_* promoter | This study |
| pEOK112 | oriV-trfA derivate, Apra^r^, *epoK and fdr_7100* with the P*_kan_* promoter | This study |
| pEOK113 | oriV-trfA derivate, Apra^r^, *epoK and fdr_S2240* with the P*_kan_* promoter | This study |
| pEOK114 | oriV-trfA derivate, Apra^r^, *epoK* and *fdx_0135*-*fdr_0130* with the P*_kan_* promoter | This study |
| pEOK115 | oriV-trfA derivate, Apra^r^, *epoK*, *fdx_0135* and *fdr_7100* with the P*_kan_* promoter | This study |
| pEOK116 | oriV-trfA derivate, Apra^r^, *epoK*, *fdx_S4580* and *fdr_S2240* with the P*_kan_* promoter | This study |
| pEOK117 | oriV-trfA derivate, Apra^r^, phage φC31 integrase gene with the P_c_ promoter, template for the oriV-trfA-Aprar vector | Laboratory stock |
| pEOK201 | pUC57, Kan^r^, *fdx_S4580* and *fdr_S2240* with the P*_kan_* promoter | GENEWIZ |
| pEOK202 | pUC57, Amp^r^, *fdx_A6445* | GENEWIZ |
| pKO0135 | p15A ori, Hyg^r^, *sacB*, upstream and downstream homologous fragments of *fdx_0135*, used to delete gene *fdx_0135* | This study |
| pKO0130 | p15A ori, Hyg^r^, *sacB*, upstream and downstream homologous fragments of *fdr_0130*, used to delete gene *fdr_0130* | This study |
| pKO7100 | p15A ori, Hyg^r^, *sacB*, upstream and downstream homologous fragments of *fdr_7100*, used to delete gene *fdr_7100* | This study |
| pXL8 | p15A ori, Hyg^r^_,_ *sacB*, *Tgint*, BT1_*attB* and *attP* | Lei *et al*. [1] |

**Table. S3** List of primers used in this study

| **Primers** | **Sequence (5’-3’)** |
| --- | --- |
| 0135-F | *CAGGATGAGGATCGTACTAG*ATGACTCACGTAGTGACCGA |
| 0135-R | *TCCTGCAGCGGCCGCTACTAGTAT*GCGCGATCAGCGAACC  AGCTC |
| 2170-F | *CAGGATGAGGATCGTACTAG*ATGAGTGAAGTTTTGAAGCC |
| 2170-R | *GTATCTTCCTGGCATCTTCC*TCAGTCGGTGAAGGCGGGCT |
| 2730-F | *CAGGATGAGGATCGTACTAG*ATGCAATACGCCCCCACCGC |
| 2730-R | *TCCTGCAGCGGCCGCTACTAGTATG*TTCAGCGCAGCAGGT  AAGGG |
| 4560-F | ATGAGCTATTACGAGCGCCA |
| 4560-R | GCACGACCTTGTTGTCCATC |
| 5185-F | *CAGGATGAGGATCGTACTAG*ATGGCCTTGATGATCACTGA |
| 5185-R | *GTATCTTCCTGGCATCTTCC*GCGCCTGCCTGTAGAAGAAG |
| 6105-F | *CAGGATGAGGATCGTACTAG*ATGCCCATCATCAAGATCCT |
| 6105-R | *TCCTGCAGCGGCCGCTACTAGTATG*TGCCACGATTATCGT  GAAGC |
| S4580-F | CGCGTATTGGGATTAGCTTG |
| S4580-R | GCCCACTGCAAGCTATCAGG |
| A6445-F | *CAGGATGAGGATCGTACTAG*ATGACCCACGTCGTCACCGA |
| A6445-R | CGGTACCTCGCGAATGCATC |
| 0130-F | ATGGAGCCCCTGCTGACGGC |
| 0130-R | TCACTCGTCGAACACCGGCG |
| 7100-F | *CAGGATGAGGATCGTACTAG*ATGAGTGCCTTCAACGAAGA |
| 7100-R | *GTATCTTCCTGGCATCTTCC*TCACTGCTCGGCAAACGCGC |
| S2240-F | GATCAACGCCCGCTTCTTCG |
| S2240-R | CGCGCCATTGGGATTCAGTC |
| test-F | GGGCATCGATCAAGAAAGGG |
| epoK-test-R | CACCGAACTGAGATACCTAC |
| 0135-test-R | GGTCGATGGCGAGGAAGTTG |
| 2170-test-R | ACATGGGCACAGCGGTTCAC |
| 2730-test-R | TAGGCGGTGCCTTTGCTGAG |
| 4560-test-R | CTTGTTGACCCGGACCTTGC |
| 5185-test-R | TCTCGATGTGCTCGGGATGG |
| 6105-test-R | GATGGCTTGGCACGACAAAC |
| S4580-test-R | ACGACTCGGGATCGATGTAG |
| A6445-test-R | TGCCGACCTTGTCCTTCCAC |
| 0130-test-R | CTGACTTCCTGGCCGTAATG |
| 7100-test-R | CTTCTCGAAGCGCTCGTAGG |
| S2240-test-R | GCGAGAGCGTGATCTCGTAG |
| 0135-0130-test-R | ACGACATGCGCCTTGATCTC |
| 0135-7100-test-R | CAGGTGCTCTTCGTAGTTGG |
| S4580-S2240-test-R | TCGAGGCCGCAGATGAAGAC |
| 0135KO-up-F | *CGGGCGGCATATCAGGATCC*GGCCAACCGATCGATTTGTC |
| 0135KO-up-R | *TGGATCACTTTCGTCAAAAA*CCTGGTCCAATTGGGTCACTAC  GTGAGTCATAC |
| 0135KO-down-F | *AACTACTCAGCACCACTAGT*CAAGCTGTCCGAGCTGGTTC |
| 0135KO-down-R | CCAGTTCGACCGAACGGATG |
| 0130KO-up-F | *CGGGCGGCATATCAGGATCC*CGCGGTTAACAAGCTCGACG |
| 0130KO-up-R | GTGCGACGACACCGTTTCAC |
| 0135KO-down-F | *AACTACTCAGCACCACTAGT*GCCCGAAGCTGCACAAGATG |
| 0135KO-down-R | GGTTGACGCTCCAGTCGAAG |
| 7100KO-up-F | *CGGGCGGCATATCAGGATCC*GAGTCCCGACATGGCCTTTG |
| 7100KO-up-R | CATGGCAGGGTGTCAGAAAG |
| 7100KO-down-F | *AACTACTCAGCACCACTAGT*TGCCGAGCAGTGACCGAAAC |
| 7100KO-down-R | CCGGTCTCCAAACGAAATAC |
| 0135-ucheck-F | ACGCTCACCGTCACGATCAG |
| 0135-ucheck-R | CGCCGGTTAAGGCTAAACTG |
| 0135-dcheck-F | CACAAATCGCCCGCAGAAGC |
| 0135-dcheck-R | CGGCTCGAGATGTTCGTAAG |
| 0135-inup-F | GGCGCAGCAGACTTGATCAC |
| 0135-inup-R | CCTTCCCGGAAACAGTCCAC |
| 0135-indown-F | AGCACTACATCCAGCTGAAC |
| 0135-indown-R | ATGCCATCGTCTTGTCCTTC |
| 0130-ucheck-F | TGCTGGTCGACATCACCAAG |
| 0130-ucheck-R | GCAAGAGATTACGCGCAGAC |
| 0130-dcheck-F | TACTCGCCGATAGTGGAAAC |
| 0130-dcheck-R | TGCGGTTGCCTTCATACACG |
| 0130-inup-F | TGCTGGTCGACATCACCAAG |
| 0130-inup-R | ATCTCCAGCAACCCGAGTTC |
| 0130-indown-F | GACACCGAGAAGTTCGAGAC |
| 0130-indown-R | CGGTAGTTGAAGCGGTTGTC |
| 7100-ucheck-F | CTTCGAGCTGTTCACCTTCC |
| 7100-ucheck-R | AGCTGGATCATCTGGATCAC |
| 7100-dcheck-F | GTGCTGGGTTGTTGTCTCTG |
| 7100-dcheck-R | TTATTGCCGCTTGCACTCCC |
| 7100-inup-F | CGCACCGCCGTCTATTTCTC |
| 7100-inup-R | GTGTCGCCCTCTTTCAGGTG |
| 7100-indown-F | AGGACCTGATCACCGAAGAG |
| 7100-indown-R | CGGCAATTCTGTAGGTTTGG |
| 0135-QF | GCTGCAAGTACACCGACTGT |
| 0135-QR | TGTAGTGCTGCTGGTTACCG |
| 0130-QF | GTGGTGGACACCGAGAAGTT |
| 0130-QR | CTGGTGGTGGTGTATTGCAG |
| 7100-QF | CTGTTCAGCTTCACGACCAC |
| 7100-QR | GCTGAAAAACTCCAGGTGCT |
| mraW-F | CGGAGGTGATACGCCACTAT |
| mraW-R | ATCCGAAGAGCCTGAAAGGT |
| Apra-F  Apra-R | TCATCGGTCAGCTTCTCAAC  AGCGGATCAACCGAGCAAAG |

*The cleavage sites are underlined, and the homologous segments are italicized.


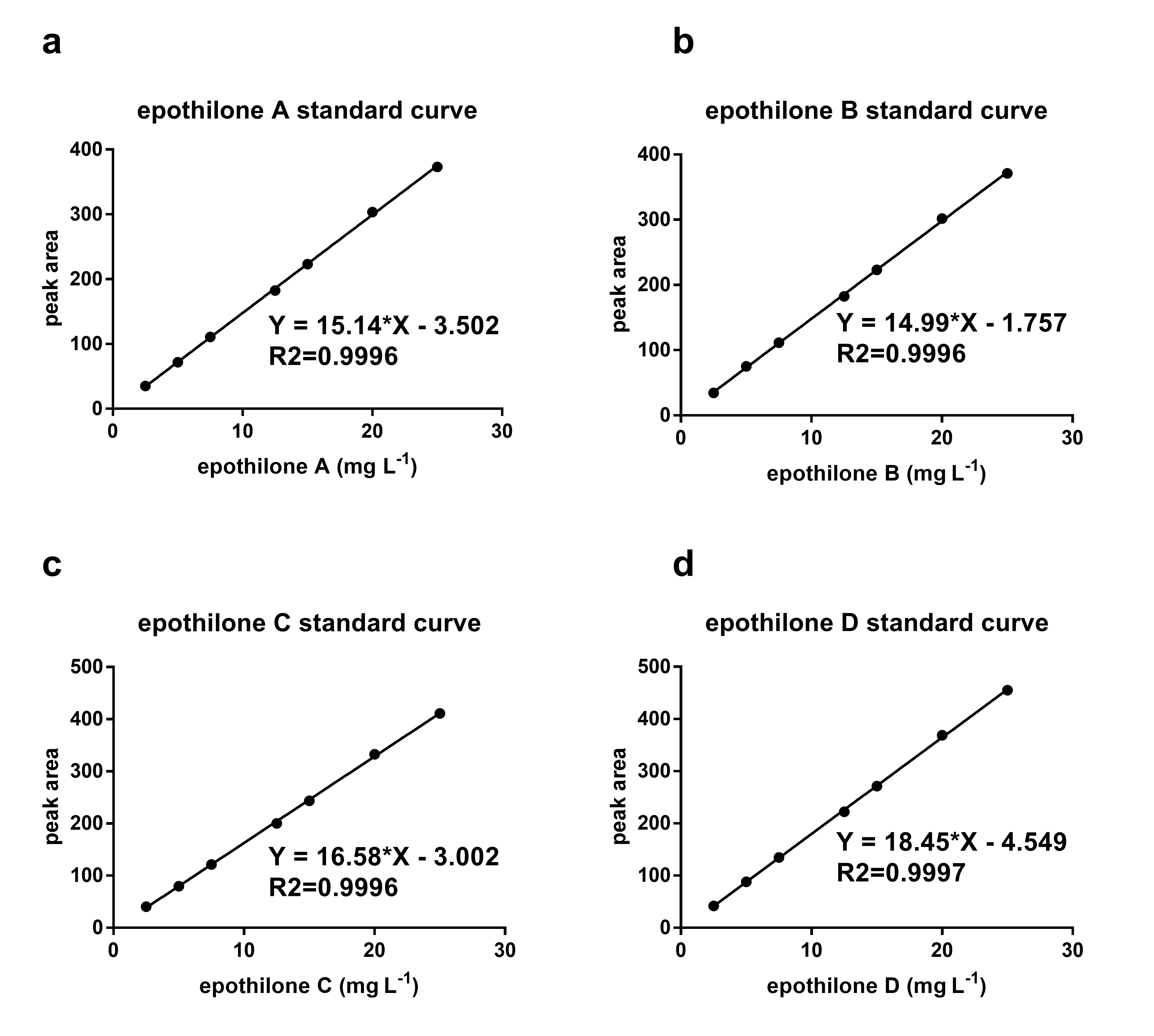


**Figure. S11. Epothilone standard sample curves calculated by the peak areas at different concentrations (2.5** **mg L**^–^**^1^, 5 mg L**^–^**^1^, 7.5 mg L**^–^**^1^, 12.5 mg L**^–^**^1^, 15 mg L**^–^**^1^, 20 mg L**^–^**^1^, and 25 mg L**^–^**^1^).**

**References:**

[1]. Lei, X., et al., Efficient circular gene knockout system for Burkholderiales strain DSM 7029 and *Mycobacterium smegmatis* mc2 155. Acta Biochimica et Biophysica Sinica, 2019. 51(7): p. 697-706.
